# Pectolinarigenin inhibits bladder urothelial carcinoma cell proliferation by regulating DNA damage/autophagy pathways

**DOI:** 10.1038/s41420-023-01508-9

**Published:** 2023-07-01

**Authors:** Zhao Deng, Dexin Shen, Mengxue Yu, Fenfang Zhou, Danni Shan, Yayun Fang, Wan Jin, Kaiyu Qian, Shenjuan Li, Gang Wang, Yi Zhang, Lingao Ju, Yu Xiao, Xinghuan Wang

**Affiliations:** 1 Department of Urology, Zhongnan Hospital of Wuhan University, Wuhan, China; 2 Department of Biological Repositories, Zhongnan Hospital of Wuhan University, Wuhan, China; 3 Human Genetic Resources Preservation Center of Hubei Province, Wuhan, China; 4 Department of Radiology, Zhongnan Hospital of Wuhan University, Wuhan, China; 5 Euler Technology, ZGC Life Sciences Park, Beijing, China; 6 Medical Research Institute, Wuhan University, Wuhan, China; 7 Center for Quantitative Biology, School of Life Sciences, Peking University, Beijing, China; 8 Hubei Key Laboratory of Urological Diseases, Wuhan University, Wuhan, China; 9 TaiKang Center for Life and Medical Sciences, Wuhan University, Wuhan, China; 10 Wuhan Research Center for Infectious Diseases and Cancer, Chinese Academy of Medical Sciences, Wuhan, China

**Keywords:** Bladder cancer, Drug development

## Abstract

Pectolinarigenin (PEC), an active compound isolated from traditional herbal medicine, has shown potential anti-tumor properties against various types of cancer cells. However, its mechanism of action in bladder cancer (BLCA), which is one of the fatal human carcinomas, remains unexplored. In this study, we first revealed that PEC, as a potential DNA topoisomerase II alpha (TOP2A) poison, can target TOP2A and cause significant DNA damage. PEC induced G2/M phase cell cycle arrest via p53 pathway. Simultaneously, PEC can perform its unique function by inhibiting the late autophagic flux. The blocking of autophagy caused proliferation inhibition of BLCA and further enhanced the DNA damage effect of PEC. In addition, we proved that PEC could intensify the cytotoxic effect of gemcitabine (GEM) on BLCA cells in vivo and in vitro. Summarily, we first systematically revealed that PEC had great potential as a novel TOP2A poison and an inhibitor of late autophagic flux in treating BLCA.

## Introduction

Bladder cancer (BLCA) is a prevalent malignancy, ranking as the sixth most common in men and one of the most common in women [1]. BLCA encompasses a range of stages, from non-muscle-invasive bladder cancer (NMIBC, approximately 70% of new BLCA diagnoses), which can recur and require long-term invasive surveillance, to muscle-invasive bladder cancer (MIBC, approximately 20% of new BLCA diagnoses), and advanced cancer (approximately 4% of new BLCA diagnoses), which has a high disease-specific mortality [2–4]. Each year, it is responsible for nearly 170,000 deaths worldwide [2]. In 2020, 573,278 new cases and 213,000 deaths of BLCA were reported by global cancer statistics, accounting for 3% of all new cases [1]. According to the latest statistics in 2022, China and the United States are estimated to have 91,893 and 84,825 new cases of BLCA, respectively. This makes BLCA the second most commonly occurring cancer of the urinary system [5, 6]. In managing BLCA, chemotherapy is the most common and basal treatment modality besides surgical resection. Intravesical treatment with chemotherapeutic agents such as Mitomycin C and GEM is used for NMIBC and neoadjuvant cisplatin-based chemotherapy is used for MIBC [3], whereas platinum-based chemotherapy remains the first-line option for advanced urothelial carcinoma [2]. However, the therapeutic options of BLCA are still limited, the options of chemotherapeutic drugs are still restricted, and there is a pressing need to discover new therapeutic agents to treat BLCA effectively.

The DNA topoisomerase family is a class of proteins that govern the topological state of the DNA in the cells. It is divided into two subfamilies: topoisomerase I (TOP1A and TOP1B) and topoisomerase II (TOP2A and TOP2B) [7]. Topoisomerases are responsible for solving various topological problems emerging along the entire length of the double-helix DNA polymer (about 3×10^9^ bp), to ensure that RNA and DNA polymerases have access to the DNA. Topoisomerases are essential for DNA replication, RNA transcription, and genome organization [8]. Additionally, the formation of irreversible topoisomerase cleavage complexes (TOPccs) can produce deleterious genomic lesions. Therefore, topoisomerases were widely exploited as targets for anti-cancer and antibacterial drugs [9]. TOPccs are well-established sources of DNA damage caused by endogenous and environmental agents. They can be trapped and stabilized by TOP2 poisons such as etoposide, doxorubicin (DOX), and mitoxantrone, with generate topoisomerase DNA-protein crosslinks (TOP-DPCs) coupled with DNA breaks. This leads to cytotoxic DNA damage and triggers DNA damage responses, such as ATR or ATM phosphorylation, p53 response, cell cycle arrest, and DNA repair mechanisms [8, 10]. High levels of TOP-DPCs have intense effects on cellular physiology [11]. TOP2 poisons could block replication and transcription by targeting TOP2, which is why the overall level of TOP2 is a critical determinant of drug sensitivity. Previous experiments have demonstrated that overexpression of TOP2A in cells increases TOP2A toxin-induced cell death, while downregulation of TOP2A decreases drug sensitivity [12, 13]. For tumors with high levels of TOP2A expression, TOP2A poisons may be highly prospective drug candidates.

Autophagy is a cellular process that plays an essential role in the degradation of longevity proteins, damaged organelles, and misfolded proteins in eukaryotic cells [14]. These capabilities allow autophagy to function primarily as a cytoprotective system [15]. Autophagy can be induced by nutrient deprivation, hypoxia, and different cellular stresses involved in cell growth, survival, and energy metabolism. Blocking autophagic flux using drugs like chloroquine (CQ) can lead to the accumulation of abnormal proteins, resulting in irreversible cell injury and cell death [16]. The use of autophagy inhibitors in cancer treatment has shown promise in clinical trials [14]. In tumor cells, DNA damage induced by various environmental or intracellular triggers specific cellular responses, including autophagy, when effective DNA damage repair is unavailable [17, 18]. DNA damage further activates several DNA damage sensors, including ATM, ATR, CHK1, CHK2, and p53, to induce cell cycle arrest, apoptosis, and cytoprotective autophagic responses [19]. Cytoprotective autophagy is one of the mechanisms of chemotherapy resistance. Hostile environment or chemotherapy drugs often induce cytoprotective autophagy, which promotes cell survival, in cancer cells through different pathways [20]. Inhibition of protective autophagy can make tumor cells sensitive to chemotherapeutic drugs [21]. This is supported by a study that showed inhibition of autophagy led to a reduction in CHK1 level, which sensitized cancer cells to DNA-damaging agents due to a significant decrease in the ability to repair DNA double-strand breaks by homologous recombination [22].

Pectolinarigenin, a flavonoid compound, was first isolated from the traditional medicinal herb, *Linaria vulgaris*. It is also the main anti-cancer active ingredient in several other plants, such as *Cirsium*, *japonicum*, *Chanroenicum*, and citrus fruits, as the main active ingredient [23]. PEC has been shown to possess anti-inflammatory properties through its dual inhibition of cyclooxygenase-2/5-lipoxygenase [24]. Previous studies demonstrated that PEC exhibits a significant inhibitory effect against hepatocellular carcinoma and gastric cancer through its suppression of the PTEN/PI3K/AKT and can cause G2/M phase cell cycle arrest, apoptosis, and changes in autophagic flux [25, 26]. Additionally, PEC has been shown to affect tumor cell survival by inhibiting the STAT3 signaling pathway in osteosarcoma, melanoma, colorectal carcinoma, and breast cancer [27–30]. However, its role in BLCA remains unclear.

Here, we demonstrate that PEC functions as a potential TOP2A poison, inducing severe DNA damage and resulting in significant G2/M phase cell cycle arrest via p53 in BLCA. Moreover, like CQ, PEC can inhibit late-stage autophagic flux, which enhances the DNA damage effect induced by PEC and further inhibits BLCA cell survival. Combination experiments also indicated that PEC is effective in inhibiting BLCA progression in combination with GEM both in vivo and in vitro.

## Results

### PEC inhibits cells proliferation and induces remarkable G2/M phase cell cycle arrest in BLCA

**Fig. 1:**
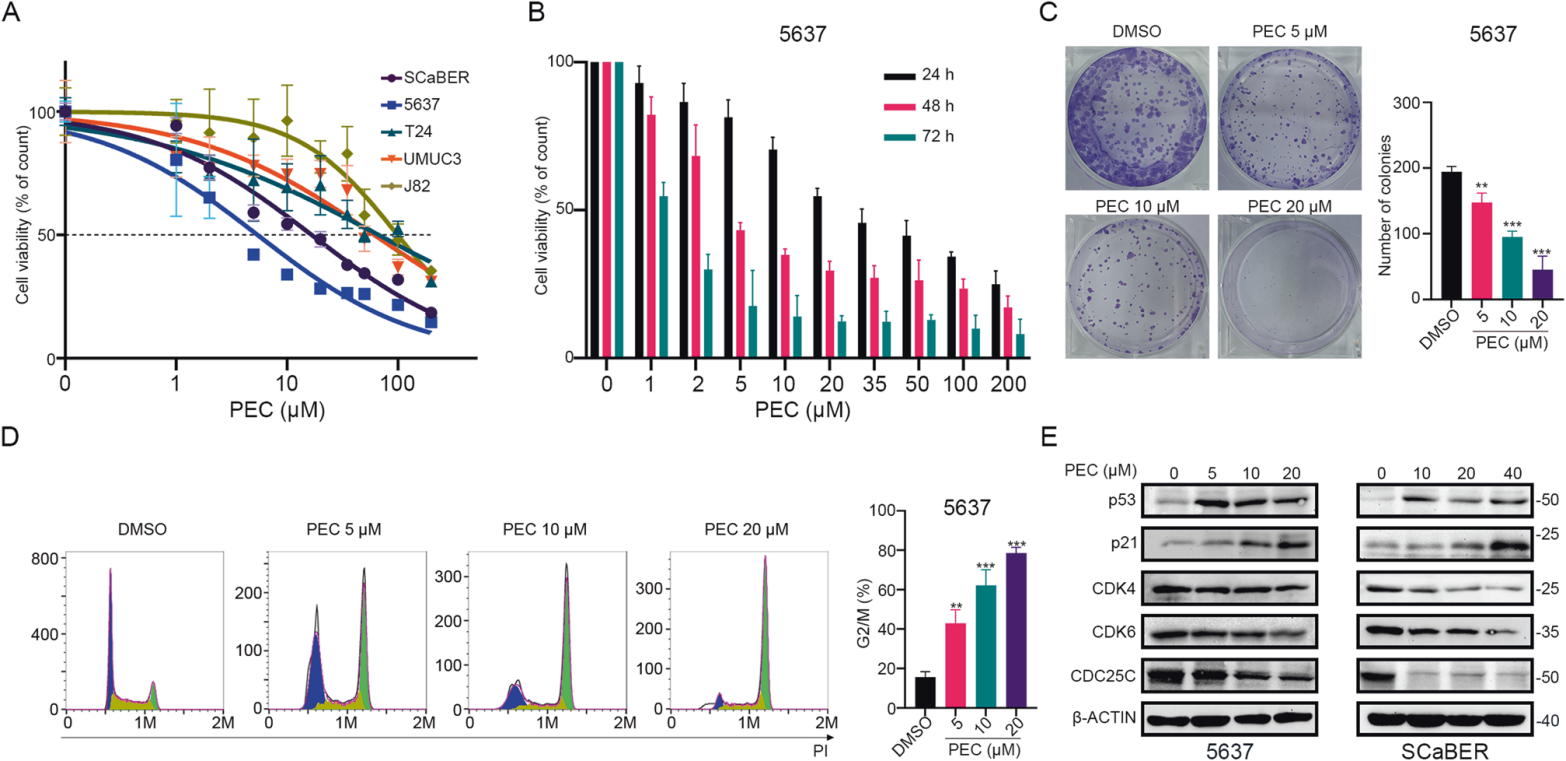
PEC inhibits cell proliferation and induces remarkable G2/M phase cell cycle arrest in BLCA. **A** The MTT assay of 5637, SCaBER, T24, UMUC3, and J82 cells treated with various concentrations (0, 1, 2, 5, 10, 20, 35, 50, 100, 200 μM) of PEC for 48 h. **B** MTT assay of 5637 cells treated with indicated concentrations of PEC for different durations (24 h, 48 h, 72 h). **C** Clone formation assay showing cell proliferation in 5637 cells treated with different concentrations of PEC. **D** Flow cytometry analysis of the cell cycle in 5637 cells treated with indicated concentrations of PEC for 24 h. **E** Western blots showing levels of proteins related to cell cycle arrest (p53, p21, CDK4, CDK6, CDC25C) in BLCA cells after being treated with PEC for 24 h.

PEC, a small molecule compound extracted from traditional herbal medicine, has been shown to have anti-tumor properties in several malignancies [23]. To explore the anti-cancer effect of PEC on BLCA cell lines, we measured its 48 h IC_50_ (half maximal inhibitory concentration) in five BLCA cell lines (the value of IC_50_: 5637 = 5.72 ± 0.39 μM; SCaBER = 17.55 ± 1.26 μM; T24 = 70.10 ± 6.02 μM; UMUC3 = 55.68 ± 14.79 μM; J82 = 136.02 ± 32.62 μM) (Fig. 1A). Our analyses revealed that while the 5637 cell line showed the highest sensitivity to PEC, the 48 h IC_50_ values of PEC in 5637 and SCaBER were significantly lower than those in the other three cell lines. Moreover, the MTT assay revealed a time- and dose-dependent inhibitory effect of PEC on BLCA cells (5637 and SCaBER) (Fig. 1B and Supplementary Fig. S1A). Clone formation assay showed a decrease in the number of colonies with increasing PEC concentrations (Fig. 1C and Supplementary Fig. S1B). To better investigate the cytotoxic mechanisms of PEC in BLCA cells, we subjected the cells to 24 h treatment with a series of PEC concentrations and used flow cytometry to analyze the effects on the cell cycle. A marked dose-dependent G2/M phase cell cycle arrest was observed in 5637 and SCaBER cells (Fig. 1D and Supplementary Fig. S1C). Moreover, there were precise changes in cycle-related proteins, including the upregulation of p53 and p21, which are essential for maintaining the G2 phase checkpoint in human cells [31] (Fig. 1E). In conclusion, our analyses indicate that PEC exerts a pronounced inhibitory effect on BLCA cells, and can induce G2/M phase cell cycle arrest.

### PEC causes significant DNA damage in BLCA cells

**Fig. 2:**
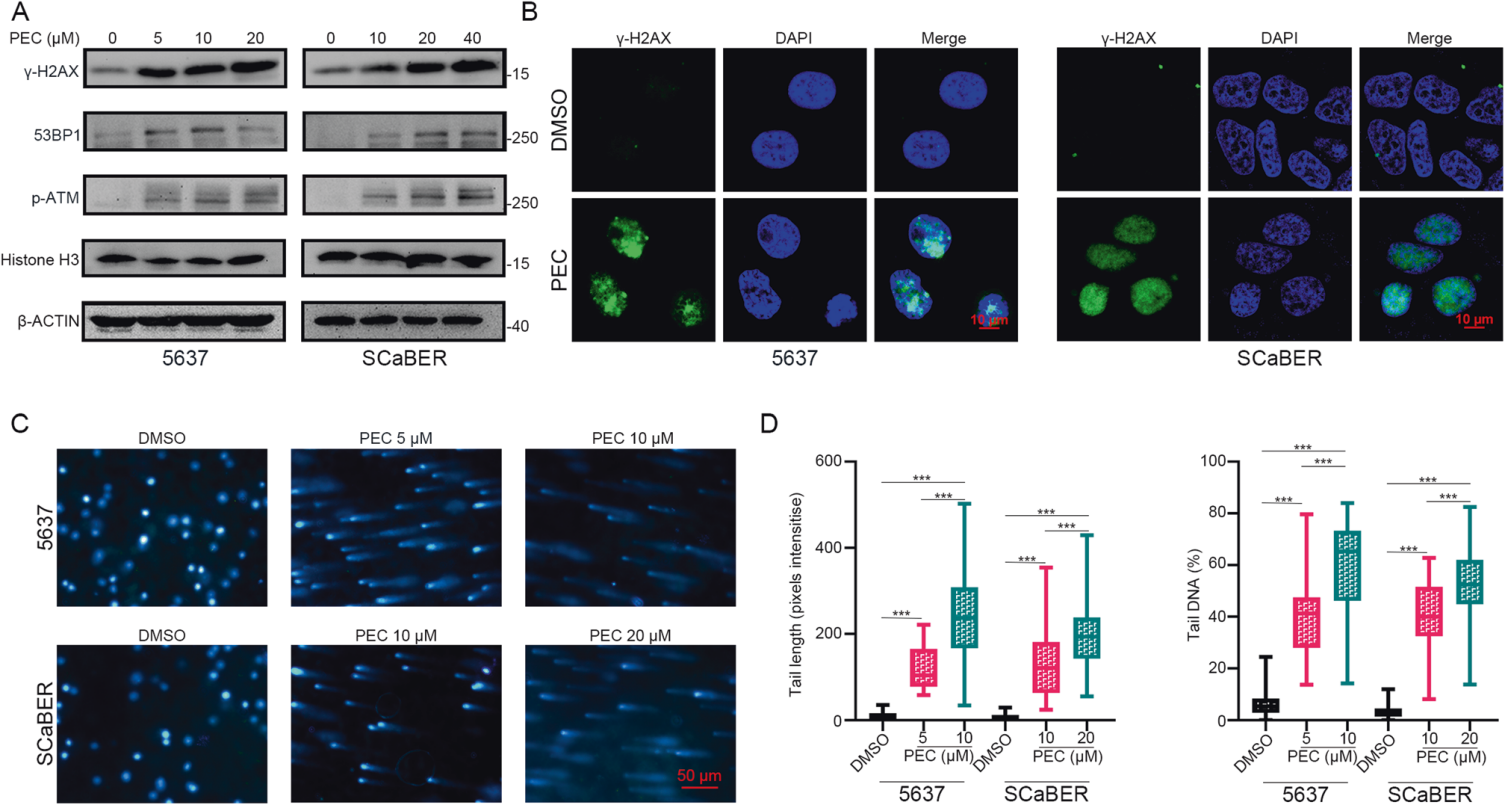
PEC causes DNA damage in BLCA cells. **A** Western blots showing proteins related to DNA damage (γ-H2AX, 53BP1, p-ATM) after PEC treatment for 24 h. **B** Immunofluorescence staining of γ-H2AX in BLCA cells after PEC treatment (5 μM in 5637, 10 μM in SCaBER) for 24 h. Scale bar, 10 μm. **C** Comet assay images showing DNA damage in the BLCA cells treated with PEC for 24 h. Scale bar, 50 μm. **D** Statistical analysis of tail length and tail DNA percentage (*n* = 50).

PEC induced a significant G2/M phase cell cycle arrest and elevation of p53 protein (Fig. 1D, E and Supplementary Fig. S1C). Previous reports have suggested that p53 is significantly associated with G2/M phase cell cycle arrest induced by DNA damage [32, 33]. Evaluating the expression levels of several important DNA damage markers, γ-H2AX, 53BP1, and p-ATM, using western blots, revealed their significant upregulation concordant with increasing PEC (Fig. 2A) [34, 35]. Moreover, immunofluorescence imaging following a PEC treatment for 24 h revealed a significant increase in γ-H2AX puncta in the cell nuclei (Fig. 2B). We further investigated the DNA breaks in BLCA cells upon PEC treatment using the comet assay, which is an effective method to assess cellular DNA damage. PEC treatment markedly increased the comet tail length and DNA percentage, indicating significant DNA damage (Fig. 2C, D). Further, we investigated the p53 dependence of PEC-induced cell cycle arrest in BLCA cells by knocking down *p53* and measuring the effect on G2/M phase cell cycle arrest. The *p*53 knockdown caused a partial reversal of the G2/M phase cell cycle arrest, indicating that the PEC-induced G2/M phase cell cycle arrest in BLCA cells partially depends on p53 activity (Supplementary Fig. S2A–C). Overall, our data show that PEC treatment causes DNA damage in BLCA cells, leading to G2/M phase cell cycle arrest through activating the p53 pathway.

### PEC exerts an anti-tumor effect by targeting TOP2A

**Fig. 3:**
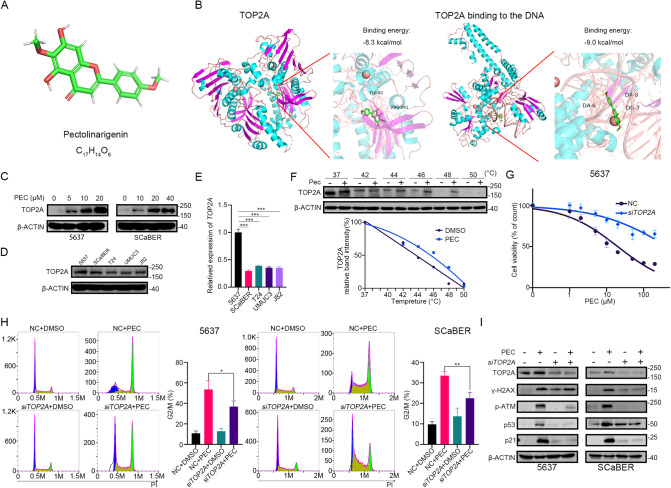
PEC exerts an anti-tumor effect by targeting TOP2A. **A** 3D structure of PEC. **B** Molecular docking of PEC and TOP2A or DNA-bound TOP2A by using AutoDockTools. **C** Western blots showing TOP2A protein level in BLCA cells incubated with indicated concentrations of PEC for 24 h. **D, E** The protein and transcript levels of TOP2A in different BLCA cell lines (5637, SCaBER, T24, UMUC3, J82). **F** Top: 5637 cell lysates were exposed for 30 min to DMSO or 100 μM PEC and subjected to CETSA. Bottom: the intensity of the TOP2A bands reflects binding affinity of PEC to TOP2A in 5637 cells. **G** MTT assay shows that knockdown *TOP2A* could reserve the inhibitory effect of PEC treatment for 48 h in 5637 cells. **H** Cell cycle analysis of 5637 and SCaBER cells transfected with *siTOP2A* or NC for 48 h, followed by treatment with or without PEC (10 μM in 5637, 20 μM in SCaBER) for 24 h. **I** Western blots showing related proteins in BLCA cells after knocking down *TOP2A* or exposing to PEC (10 μM in 5637, 20 μM in SCaBER) or combination.

As a flavone subclass compound, PEC had a typical flavone structure (Fig. 3A) [23]. Many flavones have been examined for their ability to induce DNA damage mediated by human topoisomerases. Several members of them have been shown to act as the TOP2A poisons in vivo and in vitro [36]. To investigate the potential interaction of PEC with TOP2A, we performed molecular docking analysis. Our data suggest that PEC could bind to both free TOP2A or DNA-bound TOP2A (Fig. 3B). Furthermore, the data showed that DNA-bound TOP2A had higher binding energy with PEC compared to the free TOP2A (−9.0 kcal/mol vs. −8.3 kcal/mol), suggesting that PEC binds with DNA-bound TOP2A more effectively (Fig. 3B). Western blot analysis revealed that PEC treatment for 24 h increases the protein levels of TOP2A in 5637 and SCaBER cells (Fig. 3C). Together, these data suggest that PEC acts as a potential TOP2A poison by stabilizing TOP2A-DNA cleavage complexes, which leads to an increase in the protein level of TOP2A. To investigate this effect further, we measured the RNA and protein levels of TOP2A in different BLCA cell lines (Fig. 3D, E). Consistent with this hypothesis, the 5637 cells, which showed the highest sensitivity to PEC according to the IC_50_ measurements, exhibited the highest TOP2A levels (Figs. 1A and 3D, E). Finally, we performed CETSA to assess the binding affinity of PEC towards TOP2A. We observed a shift in the TOP2A melting curve upon PEC treatment, which indicates direct binding between PEC and TOP2A protein (Fig. 3F).

TOP2A poisons bound to the TOP2A-DNA cleavage complexes cause sustained DNA break [37]. The effect of the TOP2A poisons is primarily dependent on their interaction with TOP2A, as evident from the observation that cells depleted of TOP2A are resistant to TOP2A poisons and do not show significant DNA damage. To investigate whether PEC-induced DNA damage is through its interaction with TOP2A, we knocked down *TOP2A* in 5637 and SCaBER cells using siRNA (Supplementary Fig. S3A). *TOP2A* knockdown markedly reversed the PEC-induced suppression of cell proliferation and G2/M phase cell cycle arrest in BLCA cells (Fig. 3G, H and Supplementary Fig. S3B, C). Moreover, western blot analysis revealed that *TOP2A* knockdown reversed the increasing trend of γ-H2AX and p-ATM, cycle-related proteins p53, p21 upon PEC treatment (Fig. 3I). Together, these data indicate that PEC functions as a TOP2A poison by binding to TOP2A to cause TOP2A-mediated DNA damage to suppress BLCA cell survival.

### PEC inhibits the autophagic flux in BLCA cells

**Fig. 4:**
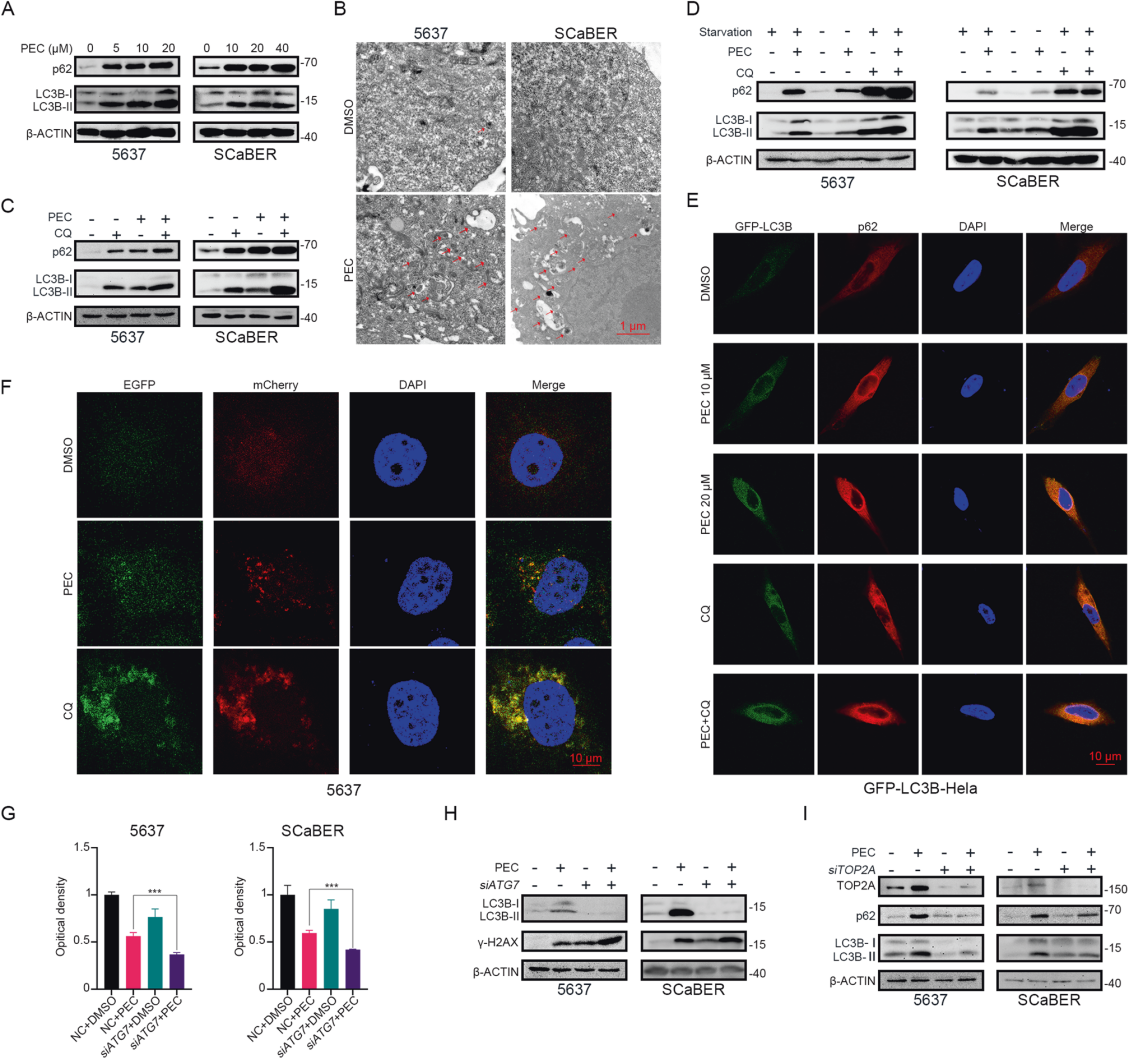
PEC inhibits the autophagic flux in BLCA cells. **A** Western blots showing protein levels of p62 and LC3B after incubation with PEC for 24 h. **B** Representative TEM images of BLCA cells exposed to PEC (10 μM in 5637, 20 μM in SCaBER) for 24 h. The red arrows mark the autophagic vacuole. Scale bar, 1 μm. **C** Western blots showing estimation of levels of p62 and LC3B in BLCA cells treated with PEC (10 μM in 5637, 20 μM in SCaBER) for 24 h and with CQ (100 μM) in the last 6 h. **D** Western blot analysis of p62 and LC3B in BLCA cells incubated with PEC (10 μM in 5637, 20 μM in SCaBER) for 24 h or CQ (100 μM) in the last 6 h or combination with serum starvation. **E** Immunofluorescence analysis of p62 and LC3B colocalization in GFP-LC3-Hela cells incubated with PEC (20 μM) or CQ (100 μM) in the last 6 h or combination. Scale bar, 10 μm. **F** Immunofluorescence analysis of mCherry-EGFP-LC3B 5637 stable cell line treated with PEC (10 μM, 24 h) or CQ (100 μM, 6 h). Scale bar, 10 μm. **G** MTT assay data showing enhancement of the inhibitory effect of PEC (10 μM in 5637, 20 μM in SCaBER) in BLCA cells upon *ATG7* knockdown by 48 h. **H** LC3B and γ-H2AX protein levels in BLCA cells transfected with *siATG7* for 48 h, followed by treatment with or without PEC (10 μM in 5637, 20 μM in SCaBER) for 24 h. **I** Western blots showing autophagy-related proteins in BLCA cells after knocking down *TOP2A* or PEC treatment (10 μM in 5637, 20 μM in SCaBER) or combination.

Autophagy is a crucial component of tumorigenesis and has become a promising therapeutic target for various cancers [38]. Although a previous study by Lee et al. showed that PEC alters the autophagic flux in gastric cancer cells, this was not confirmed in the context of BLCA [26]. Therefore, we investigated the effect of PEC on autophagy in BLCA cells. Western blot analysis revealed that PEC treatment at various concentrations resulted in a significant transformation of LC3B-I to LC3B-II in BLCA cells (Fig. 4A). TEM images also showed increased accumulation of autophagic vacuoles after PEC treatment (Fig. 4B). CQ is a classical autophagic late-stage inhibitory blocking lysosomal degradation [39]. We examined autophagy-related proteins after blocking late-stage autophagic flux by CQ. After adding CQ, we observed a further increase in LC3B-II and p62 levels in the combined group compared to the PEC-only group (Fig. 4C). Furthermore, the GFP-LC3B stable Hela cell line was used to confirm the effect of PEC. LC3B puncta increased after PEC or CQ treatment (Supplementary Fig. S4A). Together, these findings suggest that PEC induces the accumulation of autophagosomes and LC3B punctate structures in BLCA cells with the activating of autophagy.

Autophagy is a dynamic process in tumor cells comprising the formation and degradation of autophagosomes [15]. p62/SQSTM1, a cargo protein, is a marker of autophagy and its accumulation indicates inhibition of autophagy [40]. Our results showed an increase in the p62 protein levels under PEC treatment (Fig. 4A). Additionally, treatment with a combination of CQ and PEC further augmented the p62 accumulation (Fig. 4C). Moreover, PEC treatment also caused the accumulation of p62 and LC3B-II in the starvation group, in which the autophagic flux was fully activated (Fig. 4D). Treatment with PEC also increased p62 protein levels and colocalization of p62 and LC3B in GFP-LC3B cell line, similar to that observed after treatment with CQ (Fig. 4E). These results suggest a failure of autophagic degradation of p62 and LC3B-II, confirming that degradation of autophagosome was blocked after PEC treatment. To further corroborate these findings, we used two BLCA cell lines stably expressing the mCheery-EGFP-LC3B reporter probe, which can detect autophagic flux. The probe can distinguish between autophagosomes (EGFP and mCherry-positive LC3B puncta, yellow) and more acidic autolysosomes (EGFP-negative and mCherry-positive LC3B puncta, red). The results showed that the yellow puncta increased significantly after PEC treatment, similar to CQ (Fig. 4F and Supplementary Fig. S4B). These results suggest that PEC can block the autophagic flux in the late stage, similar to CQ.

To verify the effect of autophagy in BLCA cells with PEC treatment, we blocked the production of intracellular autophagy by knocking down *ATG7*, which is a key protein in the autophagy pathway. We observed enhanced cytotoxicity of PEC and DNA damage levels in BLCA cells, in which *ATG7* function was blocked (Fig. 4G-H and Supplementary Fig. S4C, D). These results suggest that PEC-induced DNA damage was not dependent on autophagic flux and inhibition of autophagy augments DNA damage. Additionally, after knocking down *TOP2A*, we observed an apparent decrease in the levels of the autophagic flux maker LC3B as well as reduced DNA damage, even under the blocking effect of CQ (Fig. 4I and Supplementary Fig. S4E). These suggest that PEC-induced DNA damage activates the initiation of autophagy.

Altogether, these results suggest that PEC influences autophagy in two ways. Firstly, PEC-induced DNA damage via TOP2A leads to an increase in autophagosome formation. Secondly, PEC ultimately blocks autophagic flux in the late stages, which enhances DNA damage and inhibition of cell proliferation.

### DOX inhibits PEC toxicity, while GEM synergistically enhances PEC cytotoxic effects

**Fig. 5:**
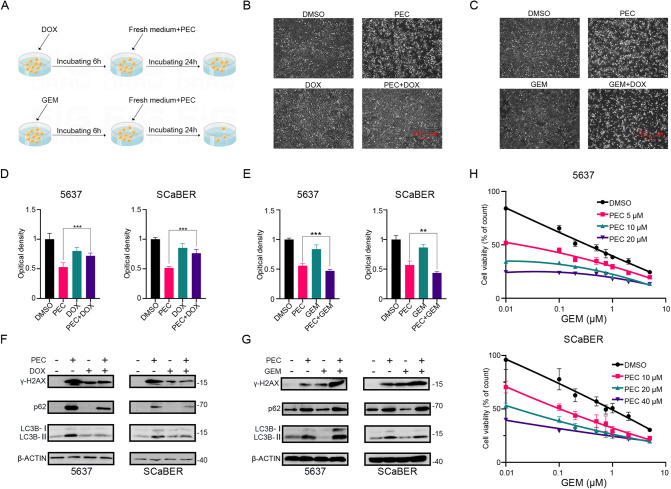
DOX inhibits PEC toxicity, while GEM synergistically enhances PEC cytotoxic effects. **A** Schematic diagram showing the experimental workflow of the drug combination assay. **B** Representative microscopic images of 5637 cells treated with DOX (250 nM, changed to fresh medium after 6 h), PEC (10 μM), or a combination. Scale bar, 500 μm. **C** Representative microscopic images of 5637 cells treated with GEM (500 nM, changed to fresh medium after 6 h), PEC (10 μM), or a combination. Scale bar, 500 μm. **D** The MTT assay of BLCA treated with DOX (250 nM, changed to fresh medium after 6 h) or PEC (10 μM in 5637, 20 μM in SCaBER) or their combination for 48 h. **E** The MTT assay of BLCA treated with GEM (500 nM, changed to fresh medium after 6 h) or PEC (10 μM in 5637, 20 μM in SCaBER) or their combination for 48 h. **F** Western blots showing protein levels in BLCA cells were treated similarly to (**B**). **G** Western blots showing protein levels in BLCA cells were treated similarly to (**C**). **H** Cell viability curves of 5637 and SCaBER cells exposed to indicated concentrations of PEC or GEM for 48 h.

DOX, a classical TOP2A poison, has been widely used in the chemotherapy of various malignancies, including BLCA [41, 42]. Our analyses showed that pretreatment with DOX (low concentration and short time) could reverse the tumor suppressive effect of PEC (Fig. 5A, B, D and Supplementary Fig. S5A). Moreover, DOX could also rescue the effect on the autophagy flux (Fig. 5F). We hypothesized that DOX (low concentration and short time) could compete with PEC to bind TOP2A, resulting in the invalidation of PEC and the reduction of DNA damage (Fig. 5F). Molecular docking analysis of DOX with TOP2A revealed that the binding energies of DOX with free TOP2A or DNA-bound TOP2A were higher than those of PEC (Supplementary Fig. S5C). Moreover, PEC and DOX could bind to the same domain and binding site of TOP2A, specifically ARG-241, further strengthening the hypothesis. Furthermore, the results of DOX or PEC docking with DNA-bound TOP2A were similar, with binding sites DG-7 and DA-8, respectively, which further confirmed our conjecture.

GEM, a classic chemotherapy drug for BLCA, is a potent and specific analog of deoxycytidine that inhibits nucleic acid synthesis. However, the cytotoxic mechanism of GEM is different from that of DOX. Moreover, GEM can induce significant DNA damage in tumor cells [43]. BLCA cells pretreated with GEM (low concentration and short time) exhibited a synergetic cytotoxic effect with PEC (Fig. 5A, C, E, and Supplementary Fig. S5B, D), characterized by augmented DNA damage levels and blockade of autophagic flux (Fig. 5G). Furthermore, 48 h treatment with a combination of PEC and GEM decreased cell growth compared to treatment with GEM alone (Fig. 5H and Supplementary Fig. S5D). These results suggest that PEC has the potential to be used in combination with GEM for the treatment of BLCA.

### PEC exerts a synergetic cytotoxic effect with GEM on BLCA cells in vivo

**Fig. 6:**
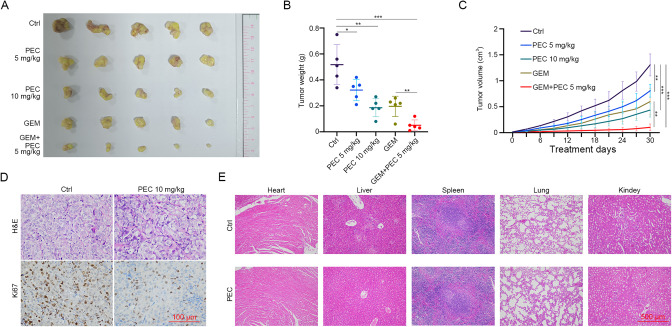
PEC exerts a synergetic cytotoxic effect with GEM on BLCA cells in vivo. **A** 5 × 10^6^ T24 cells were injected into the BALB/c-nude mice subcutaneously to construct the mouse xenograft models (*n* = 15). One week later, mice were treated intraperitoneally with control or indicated drugs. The image of dissected tumor xenografts. The weight (**B**) and volume (**C**) of tumor xenografts. **D** H&E staining and IHC of tumors from the control or PEC (10 mg/kg) group to show Ki67. Scale bar, 100 μm. **E** H&E staining of the heart, liver, lung, spleen, and kidney from the control or PEC (10 mg/kg) group. Scale bar, 500 μm.

To evaluate the anti-tumor effect of PEC and its combination with GEM in vivo, we established a mouse xenograft model of BLCA by subcutaneously injecting BLCA cells into BALB/c-nude male mice. Our results showed that PEC treatment dose-dependently suppressed tumor growth in the mouse xenograft model compared to the control group. Furthermore, the combination of PEC and GEM exhibited a more potent cytotoxic effect than that of GEM (Fig. 6A-C). IHC analysis revealed that PEC treatment significantly decreased the expression of the proliferation marker Ki67 compared to the control group (Fig. 6D). Moreover, there were no significant differences in mouse body weights between groups, demonstrating the safety of PEC treatment in vivo (Supplementary Fig. S6). Furthermore, histological assessment of morphological changes in five organ tissues (heart, liver, lung, spleen, and kidney) did not reveal any adverse effects of PEC treatment (Fig. 6E). In summary, our study demonstrates the potential of PEC as a promising therapeutic option for BLCA, both as a monotherapy and in combination with the BLCA first-line chemotherapeutic agent GEM.

## Discussion

The use of active ingredients extracted from traditional herbal medicines as therapeutic agents for cancer treatment has gained significant attention in recent years, with examples such as camptothecin, paclitaxel, and vincristine [44]. Our study shows that PEC exhibits potent anti-cancer effects against BLCA both in vivo and in vitro by inducing DNA damage and disrupting the autophagic flux. Additionally, PEC also exhibits synergistic effects with GEM in inhibiting the proliferation of BLCA cells by augmenting DNA damage. Our research provides new insights into the mechanism of action of PEC in BLCA and offers a novel therapeutic option for BLCA.

Despite the growing number of new drugs and surgical modalities implemented in the treatment of BLCA, 15-20% of NMIBC patients progress to MIBC [2, 45]. Moreover, the 5-year overall survival rate for patients with MIBC remains suboptimal, with roughly 50% eventually developing the disease at distant sites because of disseminated micro-metastases [3, 46]. Currently, the systemic treatment of MIBC and advanced BLCA consists mainly of platinum-based chemotherapies. Combination chemotherapies using cisplatin, of which the most commonly used combination is gemcitabine-cisplatin, are the standard treatment for advanced or metastatic BLCA [4, 47]. Neoadjuvant chemotherapy with cisplatin or GEM has also emerged as the standard treatment for MIBC patients [2]. For NMIBC, the preferred treatment option is the complete resection of all visible lesions in the bladder, followed either by intravesical instillations with drugs such as GEM, BCG, or Mitomycin C or early radical cystectomy for better prognosis [4, 48]. With the advances in immunotherapy, transurethral resection of bladder tumors and BCG immunotherapy have been considered the gold-standard treatment for NMIBC at high risk of recurrence or progression [48, 49]. BCG treatment triggered a strong local inflammatory response in the bladder through an immune mediator. Studies in vivo and in vitro have demonstrated that BCG treatment exerts its anti-tumor effects in BLCA through a two-step process. Firstly, it binds to fibronectin incorporated in the fibrin clot produced during transurethral resection of bladder tumors. This is followed by cytokine-mediated induction of an innate T-helper type 1 cell response, ultimately triggering an inflammatory response in the bladder. Therefore, the efficacy of BCG treatment partly depends on the formation of fibrin clots and the intensity of the inflammatory immune response within the bladder. Many studies have focused on whether there is an impact on BCG treatment when patients have to use anti-inflammatory drugs or fibrin clot inhibitors for other factors. Although the concurrent use of anti-inflammatory drugs or fibrin clot inhibitors in conjunction with BCG treatment has produced conflicting results regarding the efficacy of BCG in the treatment of NMIBC, the majority of studies currently report that anti-inflammatory drugs or fibrin clot inhibitors do not need to be discontinued during BCG treatment [50–57]. A retrospective study conducted in 2017 found that there is limited evidence to suggest that anti-inflammatory drugs, such as statins, aspirin, other NSAIDs, and COX inhibitors, have any effect on the efficacy of BCG treatment for high-risk NMIBC patients [58]. Moreover, a controlled study in 2022 suggested that the use of fibrin clot inhibitors was not associated with poor prognostic outcomes in NMIBC patients treated with BCG [59]. As mentioned above, PEC was originally validated as an anti-inflammatory agent and could inhibit COX-2-mediated PGE2 and 5-LOX-mediated LT production [24]. However, while this effect has been demonstrated in cellular and animal studies, further research is needed to determine whether it also applies to human patients with BLCA. Based on the available studies, no conflict is arising from using PEC in combination with BCG. However, it is worth noting that this would require further research into PEC’s anti-inflammatory properties, as well as its efficacy in combination with BCG. This highlights one of the limitations of this paper.

Most chemotherapy drugs kill tumor cells by causing DNA damage through various mechanisms. Our study shows that similar to most classical and curative chemotherapy drugs, PEC also causes DNA damage. Induction of DNA damage in cancer cells activates a complex network of DNA damage response signals, including DNA damage repair, cell cycle blocking checkpoints, and activation of autophagy and apoptosis. It has been the prime strategy for cancer therapy in recent decades [60, 61]. Different types of DNA damage activate specific repair mechanisms. The most common form of DNA damage, double-strand breaks, is typically repaired by homologous recombination and non-homologous end joining. This process activates ATM, ATR, and the phosphorylation of p53, CHK1, and other cycle-related proteins, leading to cell cycle arrest [62–64]. p53, a central molecule in the DNA damage response, regulates several cellular pathways, such as cell cycle arrest, apoptosis, and senescence, in response to DNA damage-induced stress. If the DNA damage is mild, the p53-mediated pathway can repair DNA damage by inducing cell cycle arrest, allowing time for DNA repair to occur before the damaged cells continue to divide. If the damage is severe and cannot be repaired, p53 could promote apoptosis, senescence, or differentiation, triggering cell death to eliminate the damaged cells from the body [65]. Therefore, even though acute DNA damage leads to p53 activation and cell cycle arrest, it has dual roles as both a killer and healer via the induction of apoptosis and DNA repair, respectively. This highlights the crucial role of the p53 protein as a gene guardian. In addition, p53 is one of the most common mutations in urothelial carcinoma [66]. The BLCA cell lines we selected, 5637 and SCaBER, carry a missense mutation in the p53 exon. Mutant p53 proteins may exhibit functions that differ significantly from their wild-type counterparts, potentially due to altered target gene profiles, structural abnormalities in the mutant protein, or improper protein-protein interactions [67]. Some p53 mutants not only lose their cancer suppressive function but also gain the ability to promote cancer cell survival relative to wild-type p53. For instance, studies have shown that overexpression of p53 mutants in lung adenocarcinoma cells can impact apoptosis, promote cell proliferation, and increase resistance to chemotherapy [68–70]. In vivo experiments have also demonstrated that transferring mutant p53 into L12 cells (lacking the cellularly encoded p53) enhances cell proliferation in mice [71]. Therefore, while wild-type p53 is recognized as a tumor suppressor, certain p53 mutants linked to cancer may possess oncogenic properties that facilitate pro-survival functions and augment chemoresistance. In our study, the knockdown of *p53* reversed the G2/M cell cycle arrest caused by PEC treatment in BLCA cells. This suggests that p53 may play a pro-survival role in response to PEC toxicity by inducing G2/M phase cell cycle arrest and subsequent cascade reactions. Of course, the mechanisms of pro-survival and chemoresistance caused by p53 mutants in BLCA cell lines still need further investigation.

Further prediction and experimental validation revealed TOP2A as a target of PEC. PEC functions as a TOP2A poison, cross-linking with irreversible TOPccs in cells to produce high levels of TOP-DPCs, ultimately leading to sustained DNA damage. TOP2A is significantly upregulated in BLCA tissues, especially in high-grade and advanced tumors [72]. Previous studies have shown that *TOP2A* expression levels were the main determining factors of tumor cell response to TOP2A poisons [13]. This was consistent with our results, where the 5637 cell line with the highest sensitivity to PEC had the highest level of *TOP2A* expression in five BLCA cell lines, suggesting that PEC may be an effective drug candidate for BLCA patients with tumors showing high *TOP2A* expression. In contrast, we found that both knockdown of *TOP2A* and low concentrations and short-term DOX treatment could reverse the cytostatic effect of PEC. DOX is a recognized TOP2A poison and a classical chemotherapy drug in BLCA [73]. Moreover, PEC-treated mice did not exhibit significant cardiac toxicity on the morphological level, which is a common side effect of DOX treatment (Fig. 6E) [74]. In addition, we have shown that PEC can exert a significant co-inhibitory effect with GEM, which induces DNA damage through a different mechanism than that of DOX. Although the combined effect of different chemotherapy drugs requires deeper analysis, our preliminary experiments suggest that combining PEC with other TOP2A poisons like DOX should be avoided in clinical treatment.

Autophagy plays a complex role in cancer development, progression, and treatment. Autophagy can be divided into four types according to its role in cancer treatment: cytoprotective, cytotoxic, cytostatic, and nonprotective [20]. Tumor cells can induce protective autophagy to resist the effect of chemotherapeutic agents and prevent the accumulation of harmful substances [21]. Inhibition of protective autophagy has been demonstrated to increase the effectiveness of anti-cancer drugs in cancer treatment [75, 76]. At present, studies have demonstrated that administering a combination of GEM and hydroxychloroquine (HCQ) to patients with pancreatic adenocarcinoma for 31 days prior to surgery is an effective, safe, and well-tolerated treatment option [77]. A phase II randomized clinical trial of GEM in combination with HCQ and albumin-bound-paclitaxel in metastasis or advanced pancreatic adenocarcinoma patients have also demonstrated an increased pathologic response rate with the combination [78]. The changes in the autophagic flux reflect cellular homeostasis’s fluctuation [79]. Altered autophagy has been certified to induce DNA damage [80]. DNA damage also can induce an increase in the autophagic flux, whereas inhibition of autophagy can impede DNA damage repair, leading to an abnormal accumulation of DNA damage [19, 22]. Our data revealed that PEC treatment increases autophagic vesicle formation, which is associated with intracellular protective autophagy, as a response to the TOP2A-mediated DNA damage effect. Interestingly, similar to CQ, PEC had a cytotoxic late-stage autophagy inhibition effect, which leads to impaired degradation of autophagosomes. Furthermore, inhibition of autophagy by PEC causes the accumulation of DNA damage in BLCA cells. Additionally, the cytotoxic inhibition of autophagy by PEC also enhanced the chemotherapeutic toxicity of other drugs causing DNA damage, such as GEM, presumably due to the accumulation of cytotoxic stresses.

**Fig. 7:**
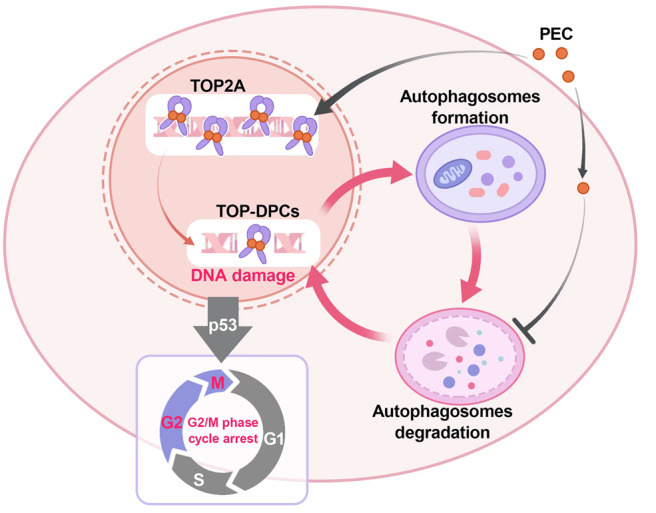
Schematic illustration of the study. PEC’s anti-cancer mechanisms against BLCA involve targeting TOP2A to induce DNA damage, G2/M phase cell cycle arrest, and inhibition of the late autophagic flux.

As concluded in Fig. 7, the current study revealed TOP2A as a novel target of PEC. PEC acts as a TOP2A poison, induces DNA damage, elicits G2/M phase cell cycle arrest, and suppresses cell growth in BLCA. In addition, PEC possessed the cytotoxic late-stage autophagy inhibition effect, which further accumulated DNA damage and suppressed cell growth. Finally, PEC shows a significant synergistic inhibitory effect on BLCA cells in combination with other chemotherapeutic agents, such as GEM.

### Statement of animal rights

For animal: this study was approved by the Experimental Animal Welfare and Ethics, Zhongnan Hospital of Wuhan University (approval No. ZN2021254).

## Materials and methods

### Cell culture and reagents

5637, SCaBER and T24 cell lines were cultured in RPMI 1640 medium. UMUC3, J82 and Hela cell lines were cultured in DMEM medium. All culture mediums were supplemented with 10% fetal bovine serum (FBS). The cell lines used in this study were purchased from the Cell Bank of the Chinese Academy of Science. GFP-LC3B Hela cell line was obtained from Prof. Min Wu (College of Life Science, Wuhan University, China). All cell lines were authenticated and tested for mycoplasma contamination before use.

Pectolinarigenin (formula: C_17_H_14_O_6_, PubChem CID: 5320438) was obtained from Shanghai Yuan Ye Biotechnology Co. Ltd., China (purity > 98%, B20761) and was dissolved in DMSO. Gemcitabine (Selleck, S1714), Doxorubicin (Selleck, S1208) and Chloroquine (Selleck, S6999) were added to the mediums to treat BLCA cells.

### Total RNA extraction and qRT-PCR

The total RNA was extracted from the cells according to the protocol provided by the HiPure Total RNA Mini Kit (Magen, R4111-03) and collected at 4 °C. RNA concentration was measured using NanoDrop^®^ ND-2000 UV–Vis spectrophotometer. And cDNA was then synthesized using ReverTrace qPCR RT Kit with 1 μg total RNA as template. Quantitative real-time polymerase chain reaction (qRT-PCR) was performed using SYBR Green PCR Master Mix. The thermocycling protocol was taken from a previous paper by Xiong et al. [81]. The primer sequences used in the qRT-PCR are provided in Supplementary Table S1.

### Cell transfection and construction of stably expressing cell lines

The sequences of siRNA (*p53*, *ATG7*, and *TOP2A*) are provided in Supplementary Table S2. All the siRNAs were purchased from GenePharma. Lipofectamine 3000 (Invitrogen, L3000015) was used to transfect cells at 30% confluence. After 48 h, cells were collected, and knockdown efficiency was evaluated by qRT-PCR.

The mCherry-EGFP-LC3B lentivirus was purchased from GenePharma. To construct stably expressing BLCA cell lines, the BLCA cells (5637, SCaBER) were transfected with the lentivirus containing polybrene (8 µg/ml) for 24 h. After two weeks, the cells were screened for resistance to puromycin (2.5 µg/ml), and the stably expressed BLCA cell lines were obtained.

### Cell cycle analysis by flow cytometry

BLCA cells were analyzed for cell cycle distribution using flow cytometry (Beckman, USA) following incubation with 1 ml of 1× DNA Staining Solution (Multi Sciences, China) and 10 µl of permeabilization solution in the dark for 30 min.

### MTT and clone formation assay

BLCA cells (3000 cells/well) were grown in a 96-well plate and allowed to adhere. The medium was then changed to a drug-containing medium for the specified duration. The BLCA cells were incubated with 3-(4,5-dimethylthiazol-2-yl)-2,5-diphenyltetrazolium bromide (MTT, 5 mg/ml) for 4 h at 37 °C. The medium was removed and 200 µl DMSO was added to each well. The cells were shaken for 30 min, and the absorbance values were measured with a microplate reader at 540 nm.

The BLCA cells were harvested and plated into six-well plates at a density of 1000 cells/well. Following treatment with the indicated drugs for 1 day, the medium was replaced with fresh medium for additional 7 days. The formed colonies were then fixed with 4% paraformaldehyde for 30 min and stained with 0.1% crystal violet for 2 h. The colonies were counted and visualized with the ImageJ software.

### Immunoblot analysis

The total protein sample was extracted with RIPA buffer and then separated by SDS-PAGE gel electrophoresis. The protein was then electro-transferred onto PVDF membranes. The membranes were blocked with 5% fat-free milk for 2 h and then incubated in primary antibodies at 4 °C overnight. After three 10 min TBST washes, the PVDF membranes were exposed to secondary antibodies for 1 h. Protein bands were visualized using an enhanced chemiluminescence kit and captured using the BioSpectrum Gel Doc-IT2 315 Imaging System (UVP, USA). The antibodies used in this study are listed in Supplementary Table S3.

### Comet assay

The comet assay is a simple and effective method for assessing cellular DNA damage [82]. Briefly, cells were collected by centrifugation (1300 rpm, 5 min) and resuspended with PBS to a concentration of 3 × 10^6^/ml. Frosted microscope slides were dipped into hot 0.8% normal-melting-point agarose in PBS to coat the frosted area. The slides were then placed in wet boxes and at 37°C overnight. The 100 µl of cell/agarose mixed liquor (25 µl of cell suspension was mixed with 75 µl of 0.6% low-melting-point agarose in PBS) was put over the first agarose layer under a coverslip evenly and rested for 20 min at 4 °C to solidify. Coverslips were removed, and the slides were immersed in cold lysis solution (2.5 M NaCl, 100 mM EDTA, 10 mM Tris-base, pH 10 in 4 °C, 1% Triton X-100, with 10% DMSO added just before use) for 6 h at 4 °C. The glass slides were then put into a horizontal electrophoresis tank filled up to about 0.25 cm above the slides with chilled electrophoresis buffer (300 mM NaOH, 1 mM EDTA, pH > 13) and left for 30 min at 4 °C to allow unwinding of DNA before electrophoresis. Electrophoresis was conducted for 30 min (30 V, 600 mA, 4 °C, in dark). The slides were washed with distilled water and neutralized 2–3 times for 5 min each time with 0.4 M Tris (pH 7.5). The slides were dehydrated with 50%, 70%, 80%, 90%, and 100% ethanol for 5 min in each solution. Each slide was stained with DAPI dyestuff for 20 min in the dark and then mounted with a coverslip. Finally, the slides were imaged using a fluorescent microscope. Fifty comet images from each group were analyzed, and the tail length and percent of DNA in tail were calculated with an open Comet Software.

### Cellular thermal shift assay (CETSA)

For the CETSA experiments, cultured 5637 cells were harvested and washed with PBS. The cells were resuspended in NP40 buffer (containing protease inhibitors) and were freeze-thawed three times in liquid nitrogen. Finally, the cell suspensions were centrifuged at 20,000 × *g* for 20 min at 4 °C to obtain the soluble fraction (lysate). The cell lysate was divided into two equal aliquots, one of which was treated with PEC (100 μM) and the other with DMSO for 30 min at room temperature. The lysates were divided equally into 6 portions (30 μl) and then heated individually at the indicated temperatures for 3 minutes in a thermal cycler (Bio-Rad, T100). The samples were then centrifuged at 20,000 × *g* for 20 min at 4 °C and the supernatant was boiled in loading buffer for western blot analysis [83].

### Immunofluorescence, immunohistochemistry (IHC), and Hematoxylin & Eosin (H&E) staining

For the immunofluorescence assay, BLCA cells were grown on slips, fixed with 4% paraformaldehyde for 30 min and then treated with buffer (2% BSA, 0.3% Triton X-100) for 1 h. Slips were incubated overnight at 4°C with primary antibodies p62 (Abcam, ab56416) or γ-H2AX (Abcam, ab81299), followed by incubation for 2 h with the secondary antibody (Invitrogen, Rabbit anti-Mouse IgG (H + L) Secondary Antibody, TRITC, PA1-28565; Invitrogen, Goat anti-Rabbit IgG (H + L) Cross-Adsorbed Secondary Antibody, FITC, F-2765) at 1:100 dilution, followed by incubation with DAPI at 1:1000 dilution for 20 min. Immunofluorescence photomicrographs were acquired on a confocal microscope (Nikon, C2^+^, Japan).

For H&E staining and IHC, the tissue sections from xenograft tumors were deparaffinized and rehydrated using xylene, alcohol (100%, 90%, 85%, and 75% in H_2_O), and H_2_O.

For H&E staining, sections were stained with 10% hematoxylin for 15 min and 1% eosin for 10 min. Sections were then imaged using automated slide scanning microscope (Thunder).

For IHC, the paraffin sections of mouse xenograft tumors were incubated with an anti-Ki67 antibody (Novus, NBP2‐19012) at 4 °C overnight, followed by incubation with the secondary antibody (Abcam, Goat Anti-Rabbit IgG H&L (HRP), ab205718), and DAB for 50 min each. Finally, nuclei were stained using hematoxylin. And the slides were dehydrated with alcohol grades (75%, 85%, 90%, and 100%) and xylene.

### Transmission electron microscopy (TEM)

BLCA cells were treated with DMSO or PEC for 24 h and then fixed in 2.5% glutaraldehyde in 0.1 M Phosphate Buffer for 1 h at 24 °C. Cells were then embedded in EPON resin for sectioning and then stained with uranyl acetate and lead citrate. The images of cells were captured with the transmission electron microscope (TEM, HT7700, Hitachi) at the Research Center for Medicine and Structural Biology, Wuhan University, China.

### Molecular docking

For docking of PEC and TOP2A in silico, we used the AutoDocktools 1.5.7 software. The crystal structures of TOP2A and TOP2A bound with DNA in complex (PDB 10.2210/pdb1ZXM/pdb; PDB 10.2210/pdb4FM9/pdb) were acquired from the Protein Data Bank (https://www.rcsb.org/). The structure of PEC (PubChem CID: 5320438) was downloaded from the PubChem database (https://pubchem.ncbi.nlm.nih.gov/). All the ligand and protein preparations were performed in the PYMOL program. Before docking, the water molecules in the structures were dislodged and hydrogen atoms were added to the protein. We defined docking grid box centered in the TOP2A protein structure using the Receptor Grid Generation tool in Glide, and then performed molecular docking using AutoDock Vina. The component with the highest degree of binding energy was selected and the results were visualized using PyMoL software [84].

### Xenograft mouse model

Specific pathogen-free male mice (BALB/c-nude, 4 weeks old, body weight ~20 g) were purchased from WTLH Co., Ltd (Beijing, China). The mice were accommodated in the new breeding environment for five days. T24 cells (4 × 10^6^ cells suspended in 100 µl PBS) were subcutaneously implanted into the bilateral axilla of mice. After seven days, the mice were randomly divided into five groups of three each. The mice were given an intraperitoneal injection of 100 µl corn oil or PEC (5 mg/kg, 10 mg/kg) or GEM (10 mg/kg) or GEM (10 mg/kg) plus PEC (5 mg/kg) once every three days for 30 days. The tumor size and body weight of mice were measured throughout the treatment period. Investigator and experimenters were blinded to the group allocation during the experiment.

### Statistical analysis

All in vitro experiments in the study were repeated at least three times, and representative data were selected from the repeats. Two-tailed Student’s t-test and one-way ANOVA were applied to evaluate the statistical significance of differences between the groups. Statistical analyses were performed using SPSS 26.0 and GraphPad Prism Version 8.0. All ‘center values’ represent mean data. All error bars represent Standard Deviation. Statistical significance was displayed as follows: ns = not statistically significant, *p < 0.05, **p < 0.01, ***p < 0.001.

## Supplementary information

Supplementary Figures S1-S6

Supplementary Tables S1-S3

## Data Availability

The original datasets and materials used and/or analyzed during the study are available from the corresponding author on reasonable request.

## References

[1] Sung H, Ferlay J, Siegel RL, Laversanne M, Soerjomataram I, Jemal A (2021). Global Cancer Statistics 2020: GLOBOCAN estimates of incidence and mortality worldwide for 36 cancers in 185 countries. CA Cancer J Clin.

[2] Patel VG, Oh WK, Galsky MD (2020). Treatment of muscle-invasive and advanced bladder cancer in 2020. CA Cancer J Clin.

[3] Lenis AT, Lec PM, Chamie K, Mshs MD (2020). Bladder cancer: a review. JAMA.

[4] Powles T, Bellmunt J, Comperat E, De Santis M, Huddart R, Loriot Y (2022). Bladder cancer: ESMO Clinical Practice Guideline for diagnosis, treatment and follow-up. Ann Oncol.

[5] Miller KD, Nogueira L, Devasia T, Mariotto AB, Yabroff KR, Jemal A (2022). Cancer treatment and survivorship statistics, 2022. CA Cancer J Clin.

[6] Xia C, Dong X, Li H, Cao M, Sun D, He S (2022). Cancer statistics in China and United States, 2022: profiles, trends, and determinants. Chin Med J.

[7] Champoux JJ (2001). DNA topoisomerases: structure, function, and mechanism. Annu Rev Biochem.

[8] Pommier Y, Nussenzweig A, Takeda S, Austin C (2022). Human topoisomerases and their roles in genome stability and organization. Nat Rev Mol Cell Biol.

[9] Kathiravan MK, Khilare MM, Nikoomanesh K, Chothe AS, Jain KS (2013). Topoisomerase as target for antibacterial and anticancer drug discovery. J Enzym Inhib Med Chem.

[10] Van Ravenstein SX, Mehta KP, Kavlashvili T, Byl JAW, Zhao R, Osheroff N (2022). Topoisomerase II poisons inhibit vertebrate DNA replication through distinct mechanisms. EMBO J.

[11] Nitiss JL (2009). Targeting DNA topoisomerase II in cancer chemotherapy. Nat Rev Cancer.

[12] Trinh BQ, Ko SY, Barengo N, Lin S-Y, Naora H (2013). Dual functions of the homeoprotein DLX4 in modulating responsiveness of tumor cells to topoisomerase II-targeting drugs. Cancer Res.

[13] Burgess DJ, Doles J, Zender L, Xue W, Ma B, McCombie WR (2008). Topoisomerase levels determine chemotherapy response in vitro and in vivo. Proc Natl Acad Sci USA.

[14] Levy JMM, Towers CG, Thorburn A (2017). Targeting autophagy in cancer. Nat Rev Cancer.

[15] Dikic I, Elazar Z (2018). Mechanism and medical implications of mammalian autophagy. Nat Rev Mol Cell Biol.

[16] Xu Y, Yu H, Qin H, Kang J, Yu C, Zhong J (2012). Inhibition of autophagy enhances cisplatin cytotoxicity through endoplasmic reticulum stress in human cervical cancer cells. Cancer Lett.

[17] Klionsky DJ, Emr SD (2000). Autophagy as a regulated pathway of cellular degradation. Science.

[18] Kang KB, Zhu C, Yong SK, Gao Q, Wong MC (2009). Enhanced sensitivity of celecoxib in human glioblastoma cells: Induction of DNA damage leading to p53-dependent G1 cell cycle arrest and autophagy. Mol Cancer.

[19] Rodriguez-Rocha H, Garcia-Garcia A, Panayiotidis MI, Franco R (2011). DNA damage and autophagy. Mutat Res.

[20] Bai Z, Peng Y, Ye X, Liu Z, Li Y, Ma L (2022). Autophagy and cancer treatment: four functional forms of autophagy and their therapeutic applications. J Zhejiang Univ Sci B.

[21] Silva VR, Neves SP, Santos LDS, Dias RB, Bezerra DP (2020). Challenges and therapeutic opportunities of autophagy in cancer therapy. Cancers.

[22] Liu EY, Xu N, O'Prey J, Lao LY, Joshi S, Long JS (2015). Loss of autophagy causes a synthetic lethal deficiency in DNA repair. Proc Natl Acad Sci USA.

[23] Cheriet T, Ben-Bachir B, Thamri O, Seghiri R, Mancini I (2020). Isolation and biological properties of the natural flavonoids pectolinarin and pectolinarigenin-a review. Antibiotics.

[24] Lim H, Son KH, Chang HW, Bae K, Kang SS, Kim HP (2008). Anti-inflammatory activity of pectolinarigenin and pectolinarin isolated from Cirsium chanroenicum. Biol Pharm Bull.

[25] Wu T, Dong X, Yu D, Shen Z, Yu J, Yan S (2018). Natural product pectolinarigenin inhibits proliferation, induces apoptosis, and causes G2/M phase arrest of HCC via PI3K/AKT/mTOR/ERK signaling pathway. Onco Targets Ther.

[26] Lee HJ, Venkatarame Gowda Saralamma V, Kim SM, Ha SE, Raha S, Lee WS (2018). Pectolinarigenin induced cell cycle arrest, autophagy, and apoptosis in gastric cancer cell via PI3K/AKT/mTOR signaling pathway. Nutrients.

[27] Deng Y, Zhang Q, Li Y, Wang L, Yang S, Chen X (2020). Pectolinarigenin inhibits cell viability, migration and invasion and induces apoptosis via a ROS-mitochondrial apoptotic pathway in melanoma cells. Oncol Lett.

[28] Gan C, Li Y, Yu Y, Yu X, Liu H, Zhang Q (2019). Natural product pectolinarigenin exhibits potent anti-metastatic activity in colorectal carcinoma cells in vitro and in vivo. Bioorg Med Chem.

[29] Li Y, Gan C, Zhang Y, Yu Y, Fan C, Deng Y (2019). Inhibition of Stat3 signaling pathway by natural product pectolinarigenin attenuates breast cancer metastasis. Front Pharmacol.

[30] Zhang T, Li S, Li J, Yin F, Hua Y, Wang Z (2022). Pectolinarigenin acts as a potential anti-osteosarcoma agent via mediating SHP-1/JAK2/STAT3 signaling. Biomed Pharmacother.

[31] Bunz F, Dutriaux A, Lengauer C, Waldman T, Zhou S, Brown JP (1998). Requirement for p53 and p21 to sustain G2 arrest after DNA damage. Science.

[32] Halazonetis TD, Gorgoulis VG, Bartek J (2008). An oncogene-induced DNA damage model for cancer development. Science.

[33] Wang X, Simpson ER, Brown KA (2015). p53: protection against tumor growth beyond effects on cell cycle and apoptosis. Cancer Res.

[34] Kleiner RE, Verma P, Molloy KR, Chait BT, Kapoor TM (2015). Chemical proteomics reveals a γH2AX-53BP1 interaction in the DNA damage response. Nat Chem Biol.

[35] Blackford AN, Jackson SP (2017). ATM, ATR, and DNA-PK: the trinity at the heart of the DNA damage response. Mol Cell.

[36] Ketron AC, Osheroff N (2014). Phytochemicals as anticancer and chemopreventive topoisomerase II poisons. Phytochem Rev.

[37] Delgado JL, Hsieh C-M, Chan N-L, Hiasa H (2018). Topoisomerases as anticancer targets. Biochem J.

[38] Li X, He S, Ma B (2020). Autophagy and autophagy-related proteins in cancer. Mol Cancer.

[39] Mauthe M, Orhon I, Rocchi C, Zhou X, Luhr M, Hijlkema K-J (2018). Chloroquine inhibits autophagic flux by decreasing autophagosome-lysosome fusion. Autophagy.

[40] Jiang P, Mizushima N (2015). LC3- and p62-based biochemical methods for the analysis of autophagy progression in mammalian cells. Methods.

[41] Svatek RS, Hollenbeck BK, Holmäng S, Lee R, Kim SP, Stenzl A (2014). The economics of bladder cancer: costs and considerations of caring for this disease. Eur Urol.

[42] Cagel M, Grotz E, Bernabeu E, Moretton MA, Chiappetta DA (2017). Doxorubicin: nanotechnological overviews from bench to bedside. Drug Discov Today.

[43] Mini E, Nobili S, Caciagli B, Landini I, Mazzei T (2006). Cellular pharmacology of gemcitabine. Ann Oncol.

[44] Wang Y, Chen M, Yu H, Yuan G, Luo L, Xu X (2022). The role and mechanisms of action of natural compounds in the prevention and treatment of cancer and cancer metastasis. Front Biosci.

[45] Knowles MA, Hurst CD (2015). Molecular biology of bladder cancer: new insights into pathogenesis and clinical diversity. Nat Rev Cancer.

[46] Yafi FA, Aprikian AG, Chin JL, Fradet Y, Izawa J, Estey E (2011). Contemporary outcomes of 2287 patients with bladder cancer who were treated with radical cystectomy: a Canadian multicentre experience. BJU Int.

[47] von der Maase H, Hansen SW, Roberts JT, Dogliotti L, Oliver T, Moore MJ (2000). Gemcitabine and cisplatin versus methotrexate, vinblastine, doxorubicin, and cisplatin in advanced or metastatic bladder cancer: results of a large, randomized, multinational, multicenter, phase III study. J Clin Oncol.

[48] Babjuk M, Burger M, Capoun O, Cohen D, Compérat EM, Dominguez Escrig JL (2022). European Association of Urology Guidelines on non-muscle-invasive bladder cancer (Ta, T1, and Carcinoma in Situ). Eur Urol.

[49] Pettenati C, Ingersoll MA (2018). Mechanisms of BCG immunotherapy and its outlook for bladder cancer. Nat Rev Urol.

[50] Lipsky MJ, Badalato GM, Motamedinia P, Hruby GW, McKiernan JM (2013). The effect of fibrin clot inhibitors on the immunomodulatory efficacy of Bacillus Calmette-Guérin therapy for non-muscle-invasive bladder cancer. Urology.

[51] Boorjian SA, Berglund RK, Maschino AC, Savage CJ, Herr HW (2009). Fibrin clot inhibitor medication and efficacy of bacillus Calmette-Guerin for bladder urothelial cancer. J Urol.

[52] Gee JR, Jarrard DF, Bruskewitz RC, Moon TD, Hedican SP, Leverson GE (2009). Reduced bladder cancer recurrence rate with cardioprotective aspirin after intravesical bacille Calmette-Guérin. BJU Int.

[53] Berglund RK, Savage CJ, Vora KC, Kurta JM, Cronin AM (2008). An analysis of the effect of statin use on the efficacy of bacillus calmette-guerin treatment for transitional cell carcinoma of the bladder. J Urol.

[54] Crivelli JJ, Xylinas E, Kluth LA, da Silva RD, Chrystal J, Novara G (2013). Effect of statin use on outcomes of non-muscle-invasive bladder cancer. BJU Int.

[55] Hoffmann P, Roumeguère T, Schulman C, van Velthoven R (2006). Use of statins and outcome of BCG treatment for bladder cancer. N Engl J Med.

[56] Skolarus TA, Lee EW, Virgo KS, Katz MD, Hudson MLA, Kibel AS (2009). Intravesical bacille Calmette-Guérin therapy for non-muscle-invasive bladder cancer: effects of concurrent statin therapy. J Am Coll Surg.

[57] Orsola A, Cecchini L, Bellmunt J (2007). Statins and the effect of BCG on bladder cancer. N. Engl J Med.

[58] Singla N, Haddad AQ, Passoni NM, Meissner M, Lotan Y (2017). Anti-inflammatory use may not negatively impact oncologic outcomes following intravesical BCG for high-grade non-muscle-invasive bladder cancer. World J Urol.

[59] Lobo N, Hensley PJ, Bree KK, Nogueras-Gonzalez GM, Navai N, Dinney CP (2022). Should patients with non-muscle-invasive bladder cancer discontinue fibrin clot inhibitors during bacille Calmette-Guérin?. BJU Int.

[60] Lord CJ, Ashworth A (2012). The DNA damage response and cancer therapy. Nature.

[61] Fokas E, Prevo R, Hammond EM, Brunner TB, McKenna WG, Muschel RJ (2014). Targeting ATR in DNA damage response and cancer therapeutics. Cancer Treat Rev.

[62] Otto T, Sicinski P (2017). Cell cycle proteins as promising targets in cancer therapy. Nat Rev Cancer.

[63] Moynahan ME, Jasin M (2010). Mitotic homologous recombination maintains genomic stability and suppresses tumorigenesis. Nat Rev Mol Cell Biol.

[64] Lieber MR (2010). NHEJ and its backup pathways in chromosomal translocations. Nat Struct Mol Biol.

[65] Helton ES, Chen X (2007). p53 modulation of the DNA damage response. J Cell Biochem.

[66] Tran L, Xiao J-F, Agarwal N, Duex JE, Theodorescu D (2021). Advances in bladder cancer biology and therapy. Nat Rev Cancer.

[67] Wang H, Guo M, Wei H, Chen Y (2023). Targeting p53 pathways: mechanisms, structures, and advances in therapy. Signal Transduct Target Ther.

[68] Blandino G, Levine AJ, Oren M (1999). Mutant p53 gain of function: differential effects of different p53 mutants on resistance of cultured cells to chemotherapy. Oncogene.

[69] Li R, Sutphin PD, Schwartz D, Matas D, Almog N, Wolkowicz R (1998). Mutant p53 protein expression interferes with p53-independent apoptotic pathways. Oncogene.

[70] Bergamaschi D, Gasco M, Hiller L, Sullivan A, Syed N, Trigiante G (2003). p53 polymorphism influences response in cancer chemotherapy via modulation of p73-dependent apoptosis. Cancer Cell.

[71] Wolf D, Harris N, Rotter V (1984). Reconstitution of p53 expression in a nonproducer Ab-MuLV-transformed cell line by transfection of a functional p53 gene. Cell.

[72] Zeng S, Liu A, Dai L, Yu X, Zhang Z, Xiong Q (2019). Prognostic value of TOP2A in bladder urothelial carcinoma and potential molecular mechanisms. BMC Cancer.

[73] Sonpavde G, Gordetsky JB, Lockhart ME, Nix JW (2016). Chemotherapy for Muscle-Invasive Bladder Cancer: Better Late Than Never?. J Clin Oncol.

[74] Doroshow JH (1991). Doxorubicin-induced cardiac toxicity. N Engl J Med.

[75] Xi G, Hu X, Wu B, Jiang H, Young CYF, Pang Y (2011). Autophagy inhibition promotes paclitaxel-induced apoptosis in cancer cells. Cancer Lett.

[76] Lin J-F, Lin Y-C, Tsai T-F, Chen H-E, Chou K-Y, Hwang TIS (2017). Cisplatin induces protective autophagy through activation of BECN1 in human bladder cancer cells. Drug Des Dev Ther.

[77] Boone BA, Bahary N, Zureikat AH, Moser AJ, Normolle DP, Wu W-C (2015). Safety and biologic response of pre-operative autophagy inhibition in combination with gemcitabine in patients with pancreatic adenocarcinoma. Ann Surg Oncol.

[78] Karasic TB, O'Hara MH, Loaiza-Bonilla A, Reiss KA, Teitelbaum UR, Borazanci E (2019). Effect of gemcitabine and nab-paclitaxel with or without hydroxychloroquine on patients with advanced pancreatic cancer: a phase 2 randomized clinical trial. JAMA Oncol.

[79] Levine B, Kroemer G (2008). Autophagy in the pathogenesis of disease. Cell.

[80] Galati S, Boni C, Gerra MC, Lazzaretti M, Buschini A (2019). Autophagy: a player in response to oxidative stress and dna damage. Oxid Med Cell Longev.

[81] Xiong Y, Yuan L, Chen S, Xu H, Peng T, Ju L (2020). WFDC2 suppresses prostate cancer metastasis by modulating EGFR signaling inactivation. Cell Death Dis.

[82] Braafladt S, Reipa V, Atha DH (2016). The comet assay: automated imaging methods for improved analysis and reproducibility. Sci Rep.

[83] Martinez Molina D, Jafari R, Ignatushchenko M, Seki T, Larsson EA, Dan C (2013). Monitoring drug target engagement in cells and tissues using the cellular thermal shift assay. Science.

[84] Kitchen DB, Decornez H, Furr JR, Bajorath J (2004). Docking and scoring in virtual screening for drug discovery: methods and applications. Nat Rev Drug Discov.

