# Supplementary material for: Pectolinarigenin inhibits bladder urothelial carcinoma cell proliferation by regulating DNA damage/autophagy pathways
Source: Cell Death Discov. 2023 Jul 1;9:214. doi: 10.1038/s41420-023-01508-9 (PMC10314945; doi:10.1038/s41420-023-01508-9)
Supplement: Supplementary file 1 — Supplementary Figures S1-S6 [file 41420_2023_1508_MOESM1_ESM.docx]

**Supplementary Figures S1-S6**


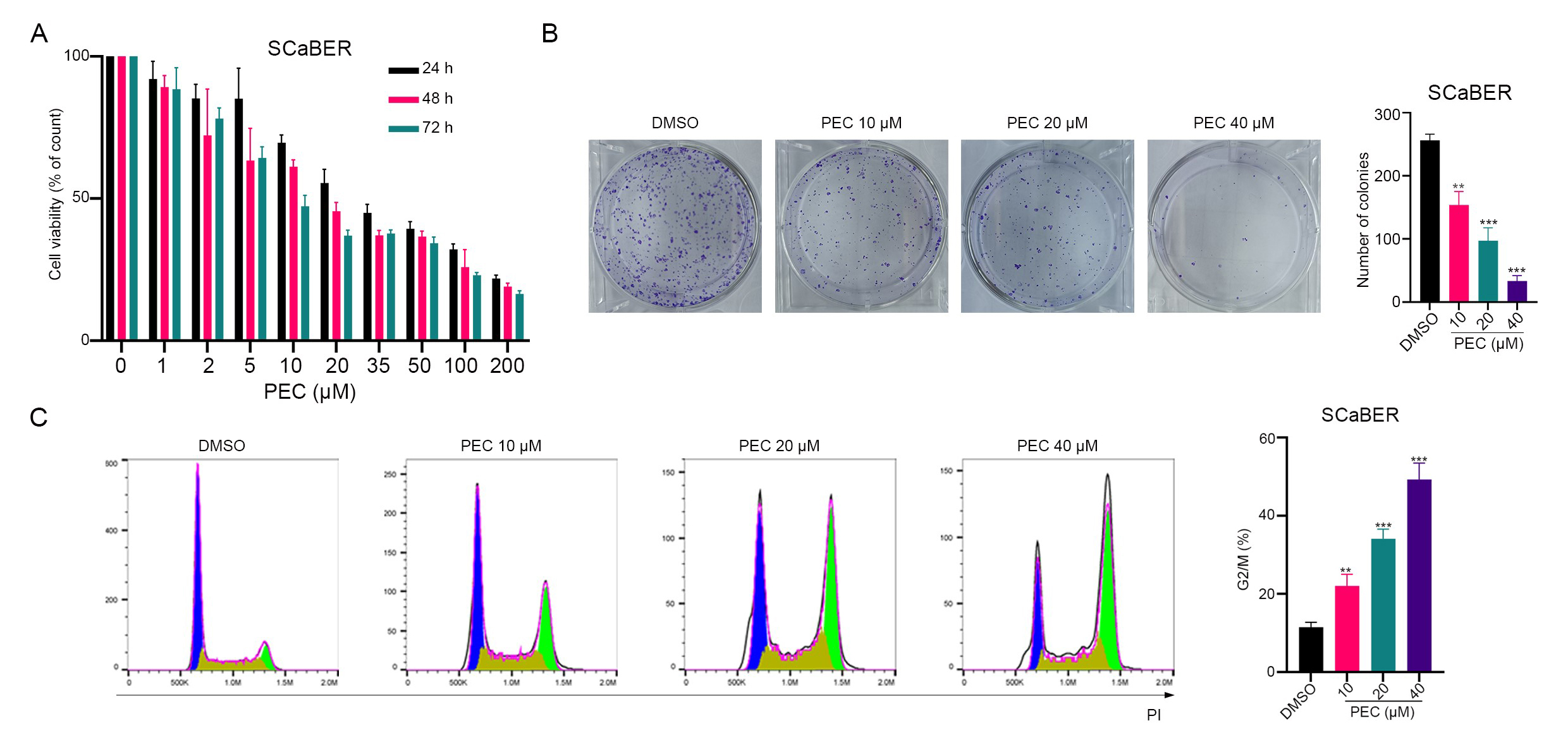


**Supplementary Figure S1.** **PEC inhibited BLCA cell proliferation and induced remarkable G2/M phase cell cycle arrest. (A)** The MTT assay of SCaBER cells was cultured with indicated concentrations of PEC and at different times (24 h, 48 h, 72 h). **(B)** Clone formation assay was used to detect the proliferation of the SCaBER cells treated with different concentrations of PEC. **(C)** Flow cytometry analysis of the cell cycle in SCaBER cells treated with indicated concentrations of PEC for 24 h.


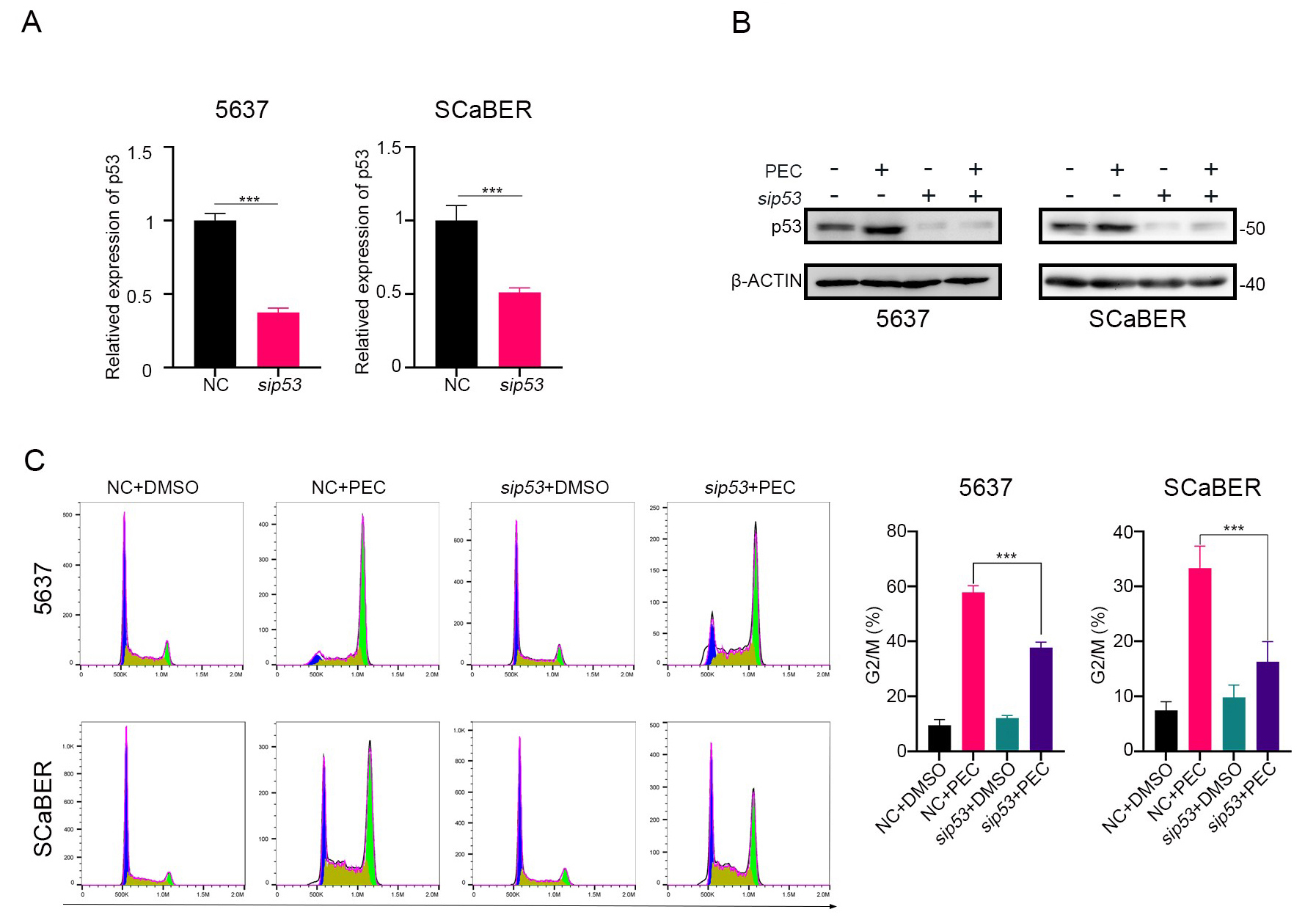


**Supplementary Figure S2. PEC resulted in significant DNA damage in BLCA cells. (A)** The efficiency of *sip53* to knockdown *p53* at the transcription level in 5637 and SCaBER is verified by qRT-PCR. **(B)** The efficiency of *sip53* to knockdown *p53* at the protein level in 5637 and SCaBER with or without PEC treatment is verified by western blots. **(C)** Cell cycle assay of BLCA cells transfected with NC or *sip53* for 48 h, followed by treatment with or without PEC (10 μM in 5637, 20 μM in SCaBER).


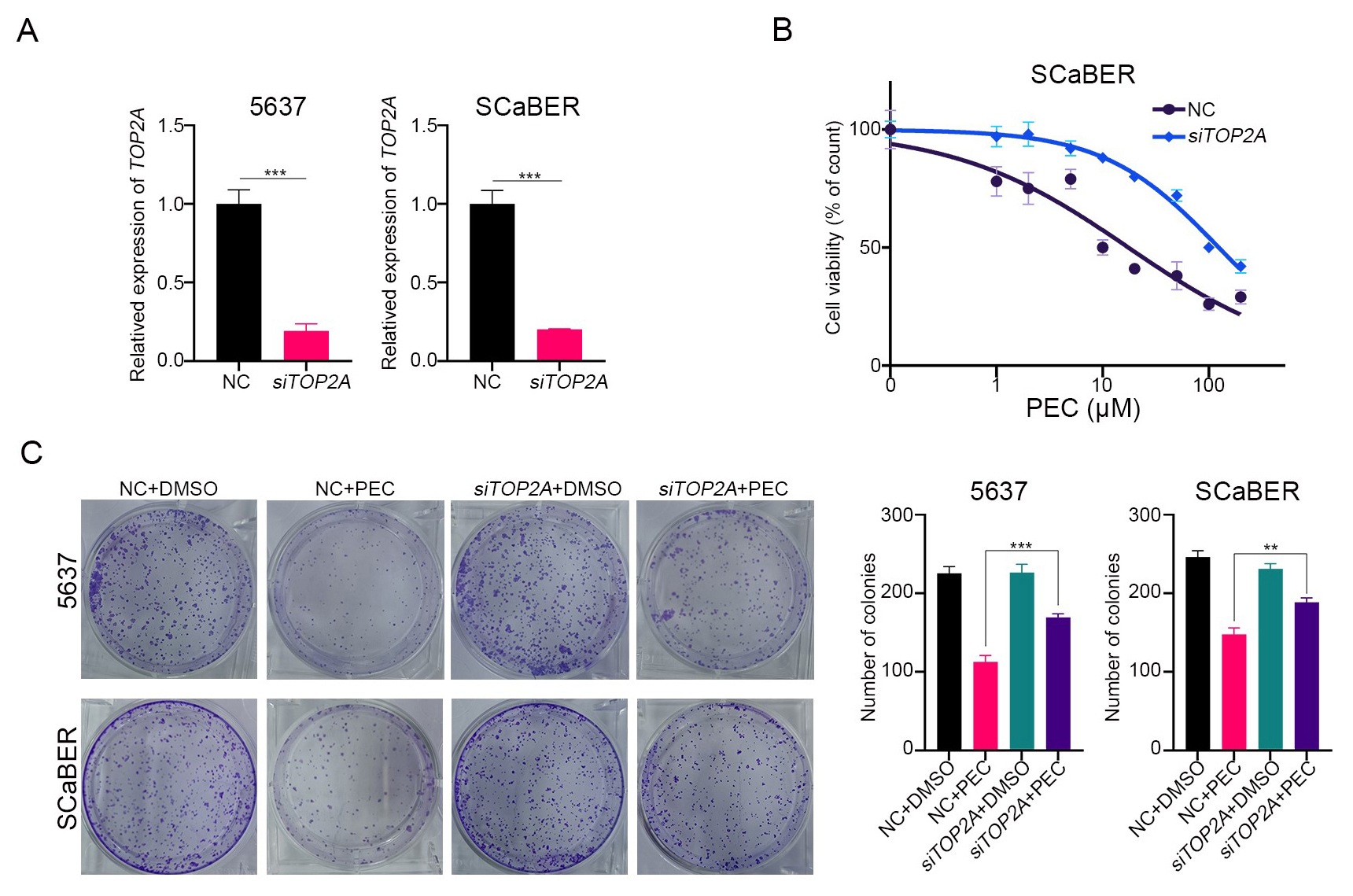


**Supplementary Figure S3.** **PEC exerted an anti-tumor effect by targeting TOP2A. (A)** The efficiency of *siTOP2A* to knockdown *TOP2A* at the transcription level in 5637 and SCaBER was verified by qRT-PCR. **(B)** The MTT assay showed that knockdown *TOP2A* could reserve the inhibitory effect of PEC treatment for 48 h in SCaBER cells. **(C)** Clone formation assay in BLCA cells transfected with NC or *siTOP2A* for 48 h, followed by treatment with or without PEC (10 μM in 5637, 20 μM in SCaBER) for 24 h.


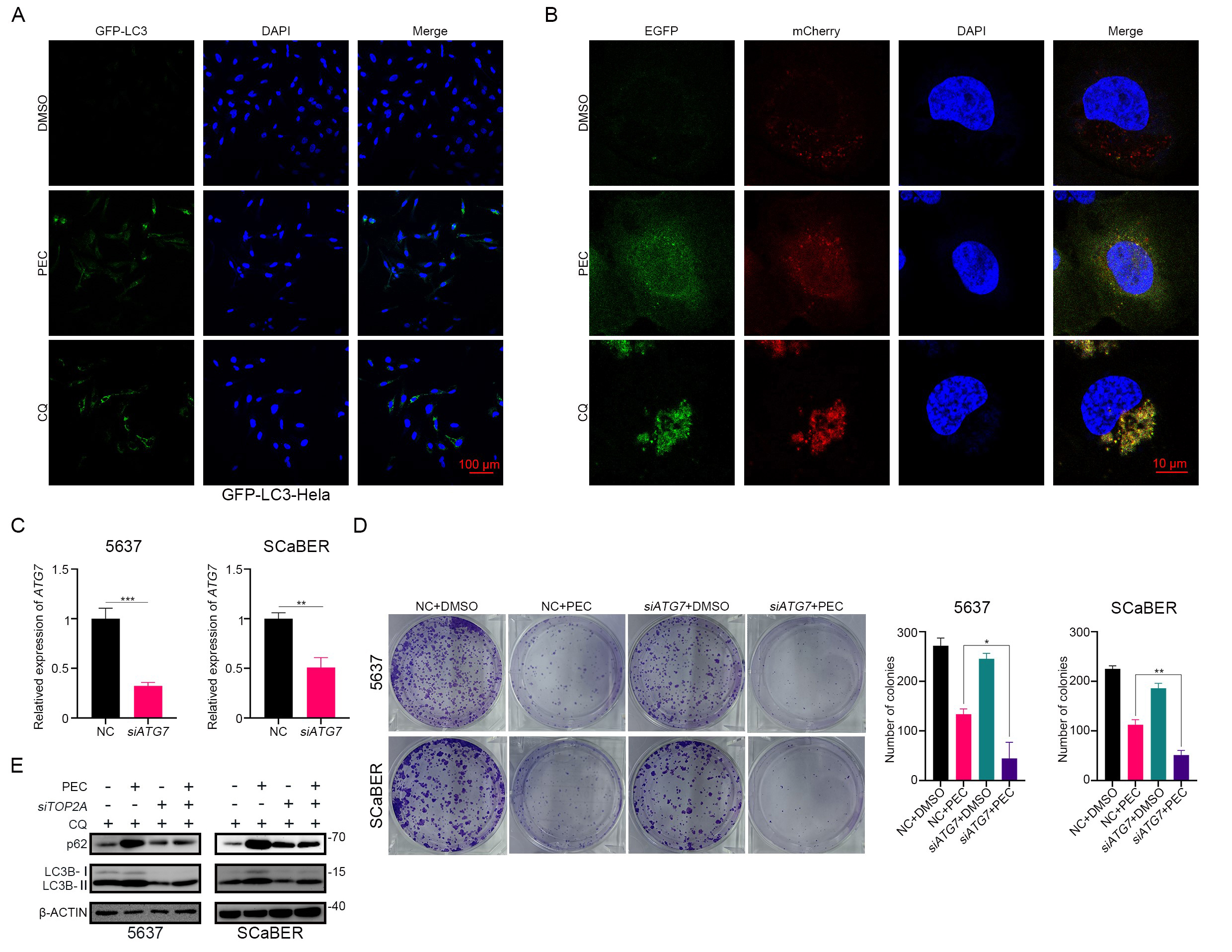


**Supplementary Figure S4. PEC inhibited autophagic flux in BLCA cells. (A)** Immunofluorescence analysis of Hela cells transfected with GFP-tagged LC3B treated with PEC 20 μM for 24 h or CQ for 6 h. Scale bar, 100 μm. **(B)** Immunofluorescence staining of SCaBER cells transfected with mCherry-EGFP-LC3B treated with PEC (20 μM, 24 h) or CQ (100 μM, 6 h). Scale bar, 10 μm. **(C)** The qRT-PCR demonstrated the efficiency of *siATG7* to knockdown *ATG7* at the transcriptional level. **(D)** Knockdown *ATG7* enhanced the inhibitory effect of PEC in BLCA cells (10 μM in 5637,20 μM in SCaBER) by clone formation assay. **(E)** LC3B and γ-H2AX protein levels in BLCA cells were transfected with *siATG7* for 48 h, followed by treatment with or without PEC (10 μM in 5637, 20 μM in SCaBER) for 24 h and all exposed to 100 μM CQ for 6 h finally.


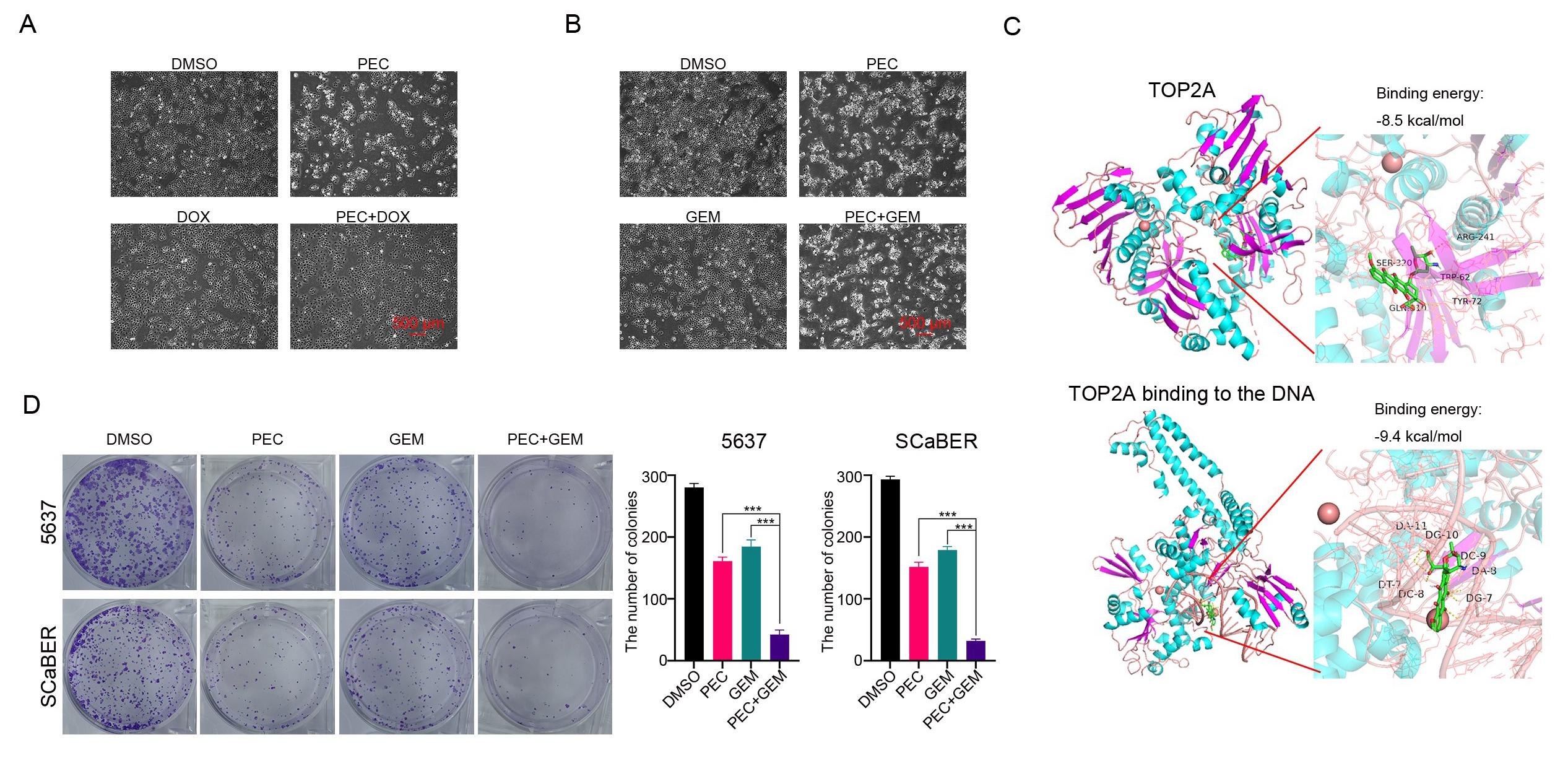


**Supplementary Figure S5. DOX inhibited PEC toxicity, while GEM synergistically enhanced PEC cytotoxic effects. (A)** Representative microscopic images of SCaBER cells treated with DOX (250 nM, changed to fresh medium after 6 h), PEC (20 μM), or combination. Scale bar, 500 μm. **(B)** Representative microscopic images of SCaBER cells treated with GEM (500 nM, changed to fresh medium after 6 h), PEC (20 μM), or combination. Scale bar, 500 μm. **(C)** Molecules docking of DOX and human TOP2A or human TOP2A bound to DNA by using AutoDockTools. **(D)** Clone formation assay in 5637 and SCaBER cells treated with PEC (10 μM in 5637, 20 μM in SCaBER) or GEM (500 nM) or in combination.

**
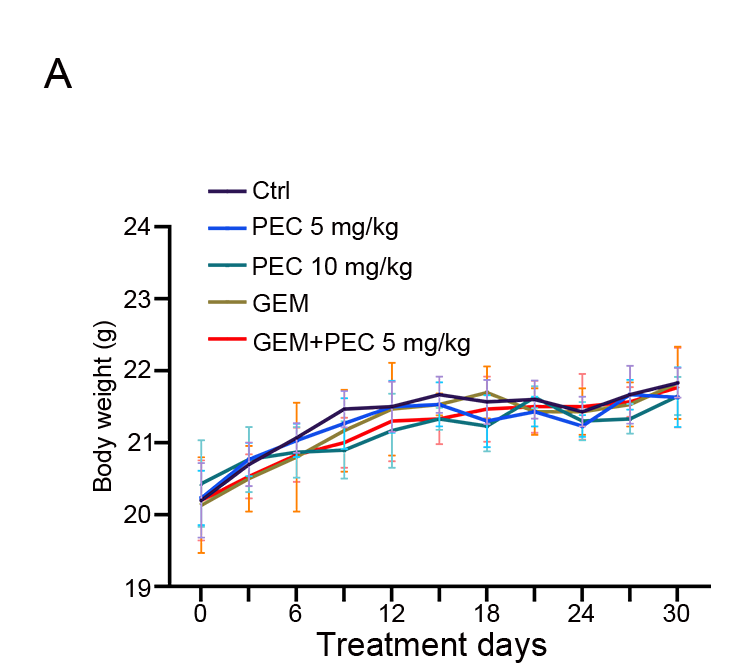
**

**Supplementary Figure S6. The combination of PEC and GEM had no effect on the body weight of the mice.** Body weight of mice, measured every three days after drug treatment.
