# Supplementary material for: Pectolinarigenin inhibits bladder urothelial carcinoma cell proliferation by regulating DNA damage/autophagy pathways
Source: Cell Death Discov. 2023 Jul 1;9:214. doi: 10.1038/s41420-023-01508-9 (PMC10314945; doi:10.1038/s41420-023-01508-9)
Supplement: Supplementary file 2 — Supplementary Tables S1-S3 [file 41420_2023_1508_MOESM2_ESM.docx]

**Supplementary Tables S1-S3**

**Supplementary Table S1. Primers for qRT-PCR.**

| **Gene** | **Forward primer (5′‐3′)** | **Reverse primer (5′‐3′)** |
| --- | --- | --- |
| *p53* | CAGCACATGACGGAGGTTGT | TCATCCAAATACTCCACACGC |
| *β-ACTIN* | GATCCACATCTGCTGGAAG | CAGCACAATGAAGATCAAGA |
| *TOP2A* | ACCATTGCAGCCTGTAAATGA | GGGCGGAGCAAAATATGTTCC |
| *ATG7* | CAGTTTGCCCCTTTTAGTAGTGC | CCAGCCGATACTCGTTCAGC |

**Supplementary Table S2. Sequences of *p53*, *ATG7*, and *TOP2A* target siRNAs.**

| **Target** | **Sequence (5′‐3′)** |
| --- | --- |
| *sip53* | GACUCCAGUGGUAAUCUAC |
| *siATG7* | GGAGTCACAGCTCTTCCTT |
| *siTOP2A* | CUCCUAACUUCUAGUAACUTT |
| NC | UUCUCCGAACGUGUCACGUTT |

**Supplementary Table S3. Antibodies used in this study.**

| **Antigens** | **Host species** | **Supplier** |
| --- | --- | --- |
| p53 | Rabbit | Proteintech, 10442-1-AP |
| p21 | Rabbit | CST, 2947 |
| CDK4 | Rabbit | CST, 12790 |
| CDK6 | Rabbit | CST, 13331 |
| CDC25C | Rabbit | Abcam, ab32444 |
| SQSTM1/p62 | Mouse | Abcam, ab56416 |
| LC3B | Rabbit | CST, 2775 |
| γ-H2AX | Rabbit | Abcam, ab81299 |
| 53BP1 | Rabbit | Abcam, ab21083 |
| p-ATM | Rabbit | Abcam, ab81292 |
| TOP2A | Rabbit | Abcam, ab52934 |
| β-actin | Mouse | Santa Cruz, sc-47778 |
| Histone H3 | Rabbit | CST, 9715 |
| Anti-Rabbit-IgG (H+L)-HRP | Goat | Jackson, Cat. #111-005-003 |
| Anti-Mouse-IgG (H+L)-HRP | Goat | Jackson, Cat. #115-005-003 |
